# Identification of a m^6^A RNA methylation regulators-based signature for predicting the prognosis of clear cell renal carcinoma

**DOI:** 10.1186/s12935-020-01238-3

**Published:** 2020-05-07

**Authors:** Jing Chen, Kun Yu, Guansheng Zhong, Wei Shen

**Affiliations:** 1 Department of Urology, Tongde Hospital of Zhejiang Province, Hangzhou, Zhejiang Province China; 2 Department of Breast and Thyroid Surgery, Zhejiang Provincial People’s Hospital, People’s Hospital of Hangzhou Medical College, Hangzhou, 310014 Zhejiang People’s Republic of China; 3 Department of Breast Surgery, The First Affiliated Hospital, College of Medicine, Zhejiang University, 79 Qingchun Road, Hangzhou, 310013 Zhejiang People’s Republic of China; 4 Department of Nephrology, Zhejiang Provincial People’s Hospital, People’s Hospital of Hangzhou Medical College, Hangzhou, 310014 Zhejiang People’s Republic of China

**Keywords:** Clear cell renal cell carcinoma, m^6^A methylation, Epigenetics, Prognostic signature, Survival analysis

## Abstract

**Background:**

The mortality rate of clear cell renal cell carcinoma (ccRCC) remains high. The aim of this study was to identify novel prognostic biomarkers by using m^6^A RNA methylation regulators capable of improving the risk-stratification criteria of survival for ccRCC patients.

**Methods:**

The gene expression data of 16 m^6^A methylation regulators and its relevant clinical information were extracted from The Cancer Genome Atlas (TCGA) database. The expression pattern of these m^6^A methylation regulators were evaluated. Consensus clustering analysis was conducted to identify clusters of ccRCC patients with different prognosis. Univariate, least absolute shrinkage and selection operator (LASSO), and multivariate Cox regression analysis were performed to construct multiple-gene risk signature. A survival analysis was carried out to determine the independent prognostic significance of the signature.

**Results:**

Five m^6^A-related genes (ZC3H13, METTL14, YTHDF2, YTHDF3 and HNRNPA2B1) showed significantly downregulated in tumor tissue, while seven regulators (YTHDC2, FTO, WTAP, METTL3, ALKBH5, RBM15 and KIAA1429) was remarkably upregulated in ccRCC. Consensus clustering analysis identified two clusters of ccRCC with significant differences in overall survival (OS) and tumor stage between them. We also constructed a two-gene signature, METTL3 and METTL14, serving as an independent prognostic indicator for distinguishing ccRCC patients with different prognosis both in training, validation and our own clinical datasets. The receiver operator characteristic (ROC) curve indicated the area under the curve (AUC) in these three datasets were 0.721, 0.684 and 0.828, respectively, demonstrated that the prognostic signature had a good prediction efficiency.

**Conclusions:**

m^6^A methylation regulators exert as potential biomarkers for prognostic stratification of ccRCC patients and may assist clinicians achieving individualized treatment for this patient population.

## Background

Kidney cancer is the sixth most commonly diagnosed cancer in men and the tenth in women, with 65,340 new cases and 16,970 deaths estimated by the latest cancer statistic report in the USA [1]. Clear cell renal cell carcinoma (ccRCC) is the most frequently primary renal cell carcinoma (RCC), accounting for approximately 70–80% of all kidney cancer [2]. Although considerable progress has been made in surgical and systemic strategies for the management of this disease, the overall survival (OS) and recurrence-free survival (RFS) is still dismal [3]. It was reported that a third of postoperative patients experienced recurrence after a median of 1.9 years [4], and regional or distant metastases further lead to a high rate of mortality [5]. Therefore, predicting prognosis of postoperative patients with high accuracy is of great significance for guiding optimal individual treatment of ccRCC. Due to the highly heterogeneity and complicated disease processes of ccRCC, there is still a lack of effective prognostic markers in this disease. Identifying novel biomarkers for predicting ccRCC patients’ long term survival is urgently needed to be addressed.

RNA modifications have been widely known to play critical roles in the post-transcriptional regulation of gene expression since its discovery in the early 1970s [6]. By the end of 2017, there were 163 different RNA modifications that have been identified in all living organisms according to MODOMICS [7]. Among these, the N^6^-methyladenosine (m^6^A) modification was identified firstly and exerted as the most abundant form of mRNA methylation, which has been found pervasively in mRNAs [8], microRNAs (miRNAs) [9], and long non-coding RNAs (lncRNAs) [10, 11]. RNA m^6^A modification, similar to traditional types of DNA and protein modification, can regulate RNA splicing, translocation, stability, and translation into protein [12, 13]. Its regulatory effects is a dynamic and reversible process [14], which is modulated by the methyltransferases called “writers” (METTL3, METTL14, WTAP, KIAA1429, RBM15, ZC3H13, and METTL16), demethylases called “erasers” (FTO and ALKBH5), and binding proteins called “readers” (YTHDF1, YTHDF2, YTHDF3, YTHDC1, YTHDC2, HNRNPA2B1, and HNRNPC) [15, 16]. Currently, accumulative evidence have demonstrated that aberrant expression of those m^6^A RNA methylation regulators is associated with multiple diseases such as infertility, obesity, and even cancer [17]. For instance, elevated METTL3 expression promotes gastric cancer progression by mediating m^6^A modification of HDGF mRNA [18]. On the contrary, YTHDF1-mediated m^6^A modification of HINT2 mRNA suppresses ocular melanoma [19]. It is clear that m^6^A methylation regulators play different or even contradictory roles in different types of cancers. Given limited knowledge of the role of m^6^A methylation in ccRCC, studying the precise correlation between m^6^A-related regulator gene and its clinical prognosis is in high demand.

In our work, we systematically analyzed the expression pattern of sixteen widely studied m^6^A related regulators in 539 tumor and 72 normal samples of ccRCC in The Cancer Genome Atlas (TCGA) datasets. Then, two clusters of ccRCC patients with different prognosis were identified through performing consensus clustering analysis. The correlation among the m^6^A RNA methylation regulators and its relationship with corresponding clinical characteristics were subsequently analyzed. Finally, based on the result of univariate, LASSO, and multivariate Cox regression analysis, we constructed and validated a two-gene risk signature by using m^6^A RNA methylation regulators, which showed a good performance to stratify the prognosis of ccRCC patients.

## Materials and methods

### Dataset acquisition

The available RNA-seq transcriptome data and clinicopathological information from 539 ccRCC samples and 72 normal samples were download from the TCGA database (https://portal.gdc.cancer.gov/). A Expectation–Maximization (RSEM) approach was used to normalize the RNA-seq data.

### Selection and differential expression analysis of m^6^A RNA methylation regulators

According to latest published review focusing on m^6^A RNA Methylation in human cancer [15, 16], we collected sixteen m^6^A RNA methylation regulators (METTL3, METTL14, WTAP, KIAA1429, RBM15, ZC3H13, METTL16, FTO, ALKBH5, YTHDF1, YTHDF2, YTHDF3, YTHDC1, YTHDC2, HNRNPA2B1, and HNRNPC) with available expression data in the TCGA datasets. The expression level of those m^6^A related genes between tumor and normal samples was compared separately by means of t-tests with a threshold of p < 0.05. Subsequently, the expression level of those genes in ccRCC with different clinical characteristics (WHO grade and AJCC stage) were also compared. Heatmaps and violin plot were utilized to visualize the different expression patterns of m^6^A related genes through “pheatmap” and “vioplot” R package.

### Consensus clustering analysis

In order to investigate the expression characteristics of m^6^A methylation regulators in ccRCC, we removed the normal tissues and clustered the tumor samples into different groups using the “ConsensusClusterPlus” R package. A principal component analysis (PCA) was subsequently conducted to verify the different gene expression patterns in different ccRCC groups. After that, the overall survival (OS) of patients in different groups was analyzed by “survival” R package. The different expression pattern of m^6^A related genes and clinicopathologic features in different groups were visualized by “pheatmap” R package. Chi square test was performed to compare the distribution of age, gender, AJCC stage, and WHO grade between the two groups. Finally, GO and KEGG pathway enrichment analysis were performed to annotate differentially expressed genes (DEGs) between different groups using the Database of Annotation, Visualization and Integrated Discovery (DAVID) v6.8 (https://david.ncifcrf.gov/), and KOBAS 3.0 (http://kobas.cbi.pku.edu.cn/). The top significantly enriched GO terms and KEGG pathways were visualized through “ggplot2” R package.

### Construction of PPI network and correlation analysis

The protein–protein interaction (PPI) network was built for these 16 m^6^A methylation regulators using the STRING online database (http://string-db.org/). Then, by means of calculating the degree of connectivity among m^6^A methylation regulators, the hub genes in PPI network were identified by using CytoHubba, a plugin in Cytoscape software (Version 3.6.1). The co-expression correlation analysis was performed to further investigate the association among those m^6^A methylation regulators, which was visualized using “corrplot” R package.

### Construction of prognostic signatures and validation

The “caret” package was used to randomly divide the samples with complete survival information into two subgroups (training group and validation group). Then, we performed univariate Cox regression analysis of the expression of m^6^A RNA methylation regulators for the training group. Genes with p < 0.05 were considered significantly associated with ccRCC patients’ survival and further selected for performing LASSO Cox regression analysis [20, 21]. Subsequently, a multivariate Cox regression analysis was conducted, and a two gene risk signature and their corresponding coefficient were finally determined. By means of multiplying the gene’s expression value and its corresponding coefficient, the risk score for each patient was calculated as the sum of each gene’s score. After that, patients were divided into high-risk and low-risk groups based on the median value of the risk score. The Kaplan–Meier method was utilized to analyze the overall survival (OS) difference between the high-risk and low-risk groups. The Receiver operating characteristic (ROC) analysis was conducted to evaluate the prediction efficiency of the two-gene risk signature. Heatmaps was utilized to visualize the different expression pattern of those two genes between high-risk and low-risk groups with “pheatmap” R package. Furthermore, this two-gene risk signature was validated in the validation group by plotting Kaplan–Meier curve and ROC curve.

### Independent prognostic ability of the multi-gene signature

Univariate and multivariate Cox regression analyses were conducted both in training and validation group to determine whether risk score and corresponding clinicopathologic features were independent prognostic factors for ccRCC patients. The Kaplan–Meier method was also applied to compare the overall survival (OS) difference between the high-risk and low-risk groups stratified by age, gender, AJCC stage, and WHO grade.

### Cell culture

The human ccRCC and immortalized proximal tubule epithelial cell line, 786-O and HK2, were purchased from the Cell Bank of the Chinese Scientific Academy. These two cell lines were maintained in Roswell Park Memorial Institute (RMPI)-1640 medium (Gibco; Life Technologies; Thermo Fisher Scientific, Inc.) and Dulbecco’s modified Eagle’s medium (DMEM) (Gibco; Life Technologies; Thermo Fisher Scientific, Inc.), respectively, with 10% fetal bovine serum (FBS; Biological Industries) at 37 °C and 5% CO_2_.

### Validation of the selected gene signatures using quantitative real-time PCR

The clinical samples were collected from ccRCC patients by experienced surgeons at the First Affiliated Hospital of Zhejiang University. The study was approved by the Institute Ethics Committee of the hospital, and the written informed consents were provided from all patients. After being surgically resected, the samples were stored in liquid nitrogen.

The total RNA of the cell lines and clinical samples was extracted using Trizol reagent (Invitrogen, USA). The cDNA for each cell line and tissue samples were reverse transcribed using the PrimeScript 1st Strand cDNA Synthesis Kit (TaKaRa, Dalian, China). qRT-PCR analysis was conducted using the SYBR-Green method according to standard protocols. The sequences of the primers used were as follows: METTL3, 5′-TTGTCTCCAACCTTCCGTAGT-3′ (forward), 5′-CCAGATCAGAGAGGTGGTGTAG-3′ (reverse); METTL14, 5′-AGTGCCGACAGCATTGGTG -3′ (forward), 5′-GGAGCAGAGGTATCATAGGAAGC-3′ (reverse).

### Statistical analysis

All data in the present study were analyzed using the R statistical package (R version 3.6.1) unless otherwise stated. A two-tailed p < 0.05 was considered statistically significant.

## Results

### The expression pattern of m^6^A RNA methylation regulators in ccRCC

**Fig. 1:**
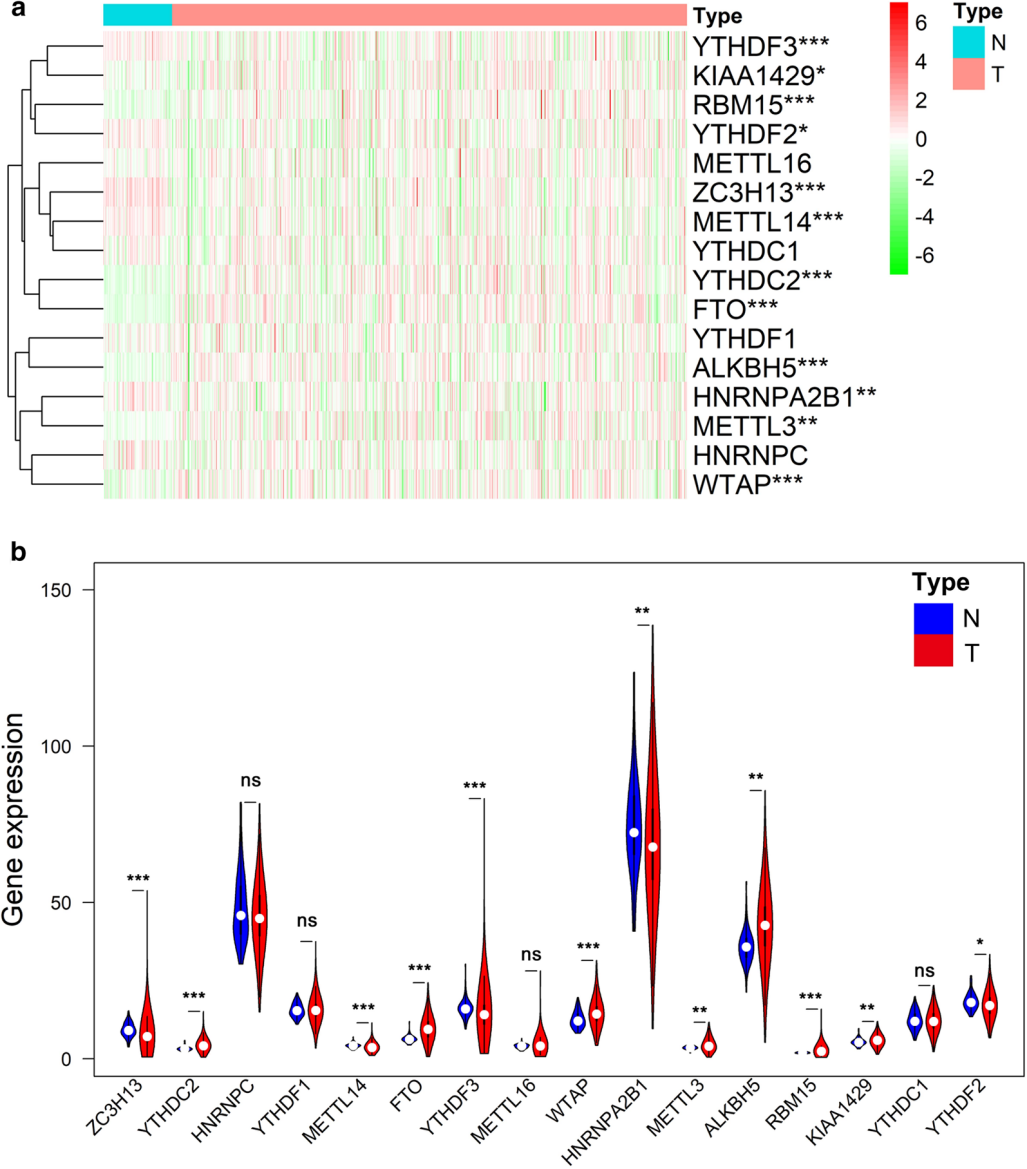
The expression pattern of 16 selected m^6^A RNA methylation regulators in TCGA ccRCC cohort. **a** Heatmap visualizing the expression levels of m^6^A RNA methylation regulators in tumor samples and normal samples. **b**Vioplot visualizing the differentially expressed m^6^A RNA methylation regulators in ccRCC. *p < 0.05, **p < 0.01, ***p < 0.001

In order to explore the expression pattern of m^6^A RNA methylation regulators in ccRCC, the sequencing data of a total of 16 m^6^A related genes were extracted from the TCGA ccRCC cohort. Compared with 72 normal kidney tissue in TCGA dataset, 5 out of 16 genes (ZC3H13, METTL14, YTHDF2, YTHDF3 and HNRNPA2B1) showed significantly lower expression level (p < 0.05) in 539 ccRCC tissues, while 7 genes (YTHDC2, FTO, WTAP, METTL3, ALKBH5, RBM15 and KIAA1429) represented relatively high expression (p < 0.05) (Fig. 1a and b). No significant difference was found for METTL16, YTHDC1, YTHDF1 and HNRNPC (p > 0.05).

### The interaction and correlation among the m^6^A RNA methylation regulators in ccRCC

**Fig. 2:**
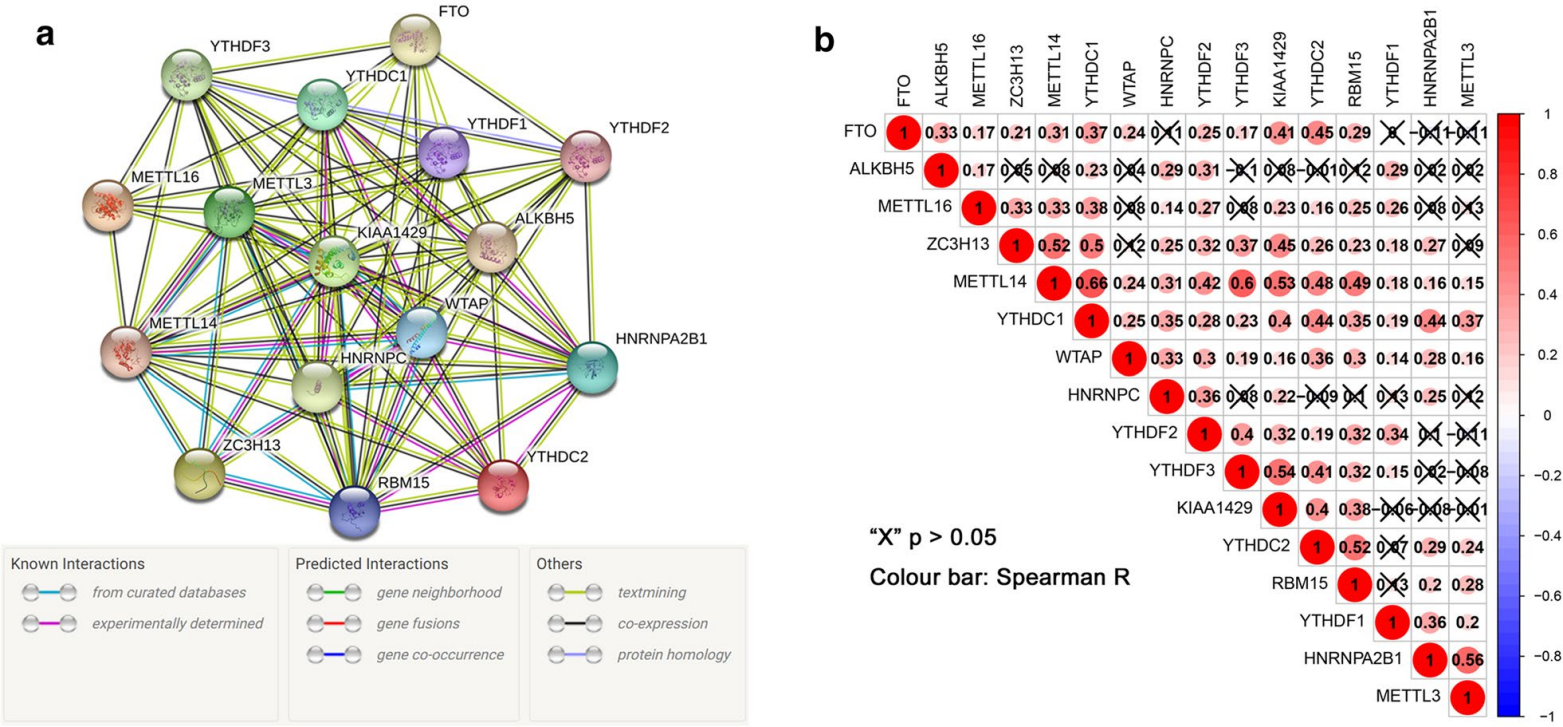
The interaction and correlation among 16 selected m^6^A RNA methylation regulators. **a** The protein–protein (PPI) network of the 16 selected m^6^A RNA methylation regulators; **b** The Pearson correlation analysis of the 16 selected m^6^A RNA methylation regulators in TCGA ccRCC cohort

In order to understand the mutual interaction of the m^6^A RNA methylation regulators, the PPI network of the chosen 16 genes was constructed based on the String database. As shown in Fig. 2a, the 16 m^6^A RNA methylation regulators exhibited complicated interactions among each other. According to the node degree calculated by the cytoscape software, 3 genes (METTL3, METTL14 and KIAA1429) were identified as the hub genes (Additional file 1: Fig. S1). Moreover, the correlation analysis was conducted to analyze the interaction among these regulators in ccRCC, suggesting that part of the different m^6^A RNA methylation regulators showed weakly to moderately positive correlation (Fig. 2b). Among all the 16 regulators, YTHDC1 was correlated with all of the other m^6^A RNA methylation regulators. Except for ALKBH5, METTL14 was associated with all the other 15 regulators and had the strongest correlation with YTHDC1 (r = 0.66).

### Consensus clustering of m^6^A RNA methylation regulators identified two clusters of ccRCC with distinct clinical outcomes

**Fig. 3:**
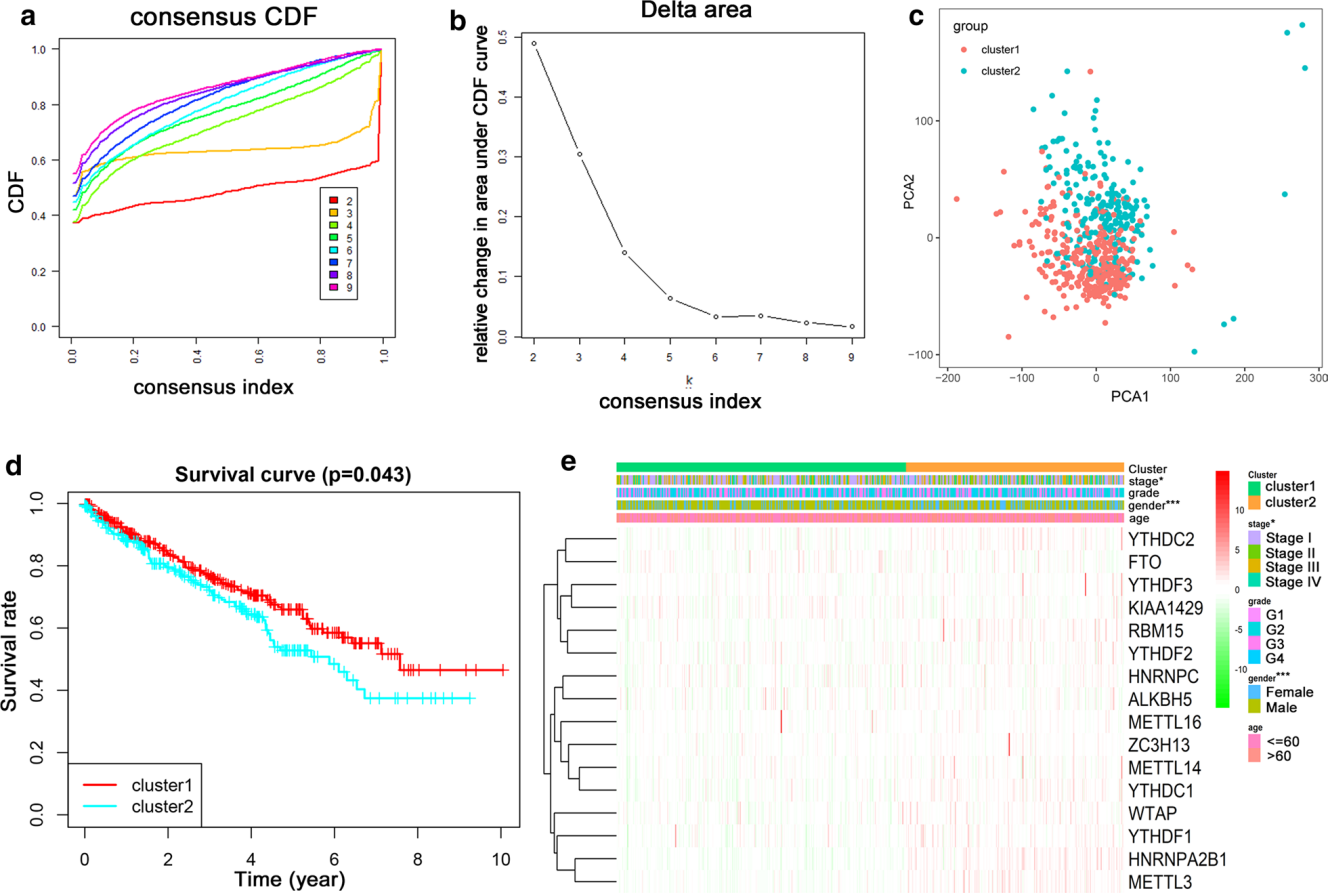
Differential expression pattern and clinical outcome of TCGA ccRCC patients in the two different clusters. **a** Consensus clustering cumulative distribution function (CDF) for k = 2–9; **b** relative change in area under CDF curve for k = 2–9; **c** Principal component analysis of the total RNA expression profile in the TCGA ccRCC cohort; **d** The survival analysis for the two clusters by Kaplan–Meier method; **e** Heatmap and clinicopathologic features of the two clusters defined by the m^6^A RNA methylation regulators consensus expression. *p < 0.05, **p < 0.01, ***p < 0.001

Based on the expression similarity of m^6^A RNA methylation regulators, we clustered the tumor samples into different groups using the ConsensusClusterPlus package. As shown in Fig. 3a and b, k = 2 seemed to be the most appropriate selection, which could divide the ccRCC cohort into two groups with acceptable stability, namely cluster 1 and cluster 2 (Additional file 2: Fig. S2). The principal component analysis (PCA) showed a clear distinction of the transcriptional profile between cluster 1 and cluster 2 subgroups (Fig. 3c). To better understand the difference between them, we identified the dysregulated genes in cluster 2 compared with cluster 1 with a threshold of adjust p < 0.05 and |log_2_FC| > 1, and then annotated its biological functions using GO and KEGG pathway analysis. The biological processes analysis in GO annotation indicated that dysregulated genes were significantly enriched in terms related with cell proliferation, such as positive regulation of cell proliferation and cell division (Additional file 3: Fig. S3a). Moreover, various cancer-related pathways were observed in KEGG pathway analysis, such as wnt signaling pathway, PPAR signaling pathway, and AMPK signaling pathway (Additional file 3: Fig. S3b).

In order to investigate the association between the clustering result and clinical outcome, we compared the overall survival (OS) of ccRCC patients between cluster 1 and cluster 2. The result indicated that the ccRCC patients in cluster 2 had a significantly shorter OS than cluster 1 (p < 0.05) (Fig. 3d). Subsequently, the relationship analysis of clinicopathological characteristics between the two clusters demonstrated significant difference for the stage and gender (p < 0.05) (Fig. 3e). These results, taken together, suggested that the clustering result was closely related to the clinical outcome and malignancy of clear cell renal cell carcinoma.

### Construction of a two-gene risk signature with distinct prognostic value

**Fig. 4:**
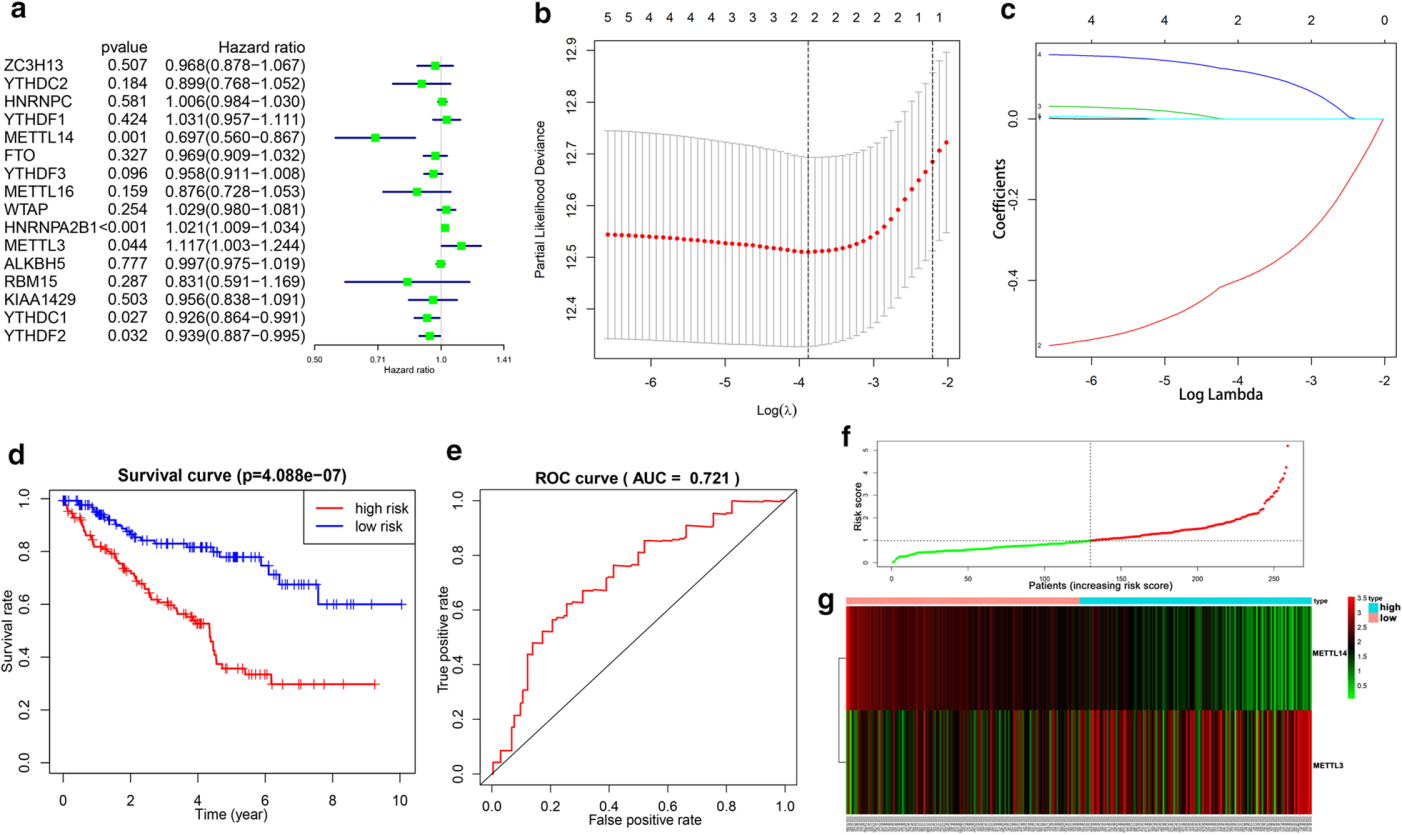
Construction of prognostic risk signature with two m^6^A RNA methylation regulators. **a** Univariate Cox analysis of the selected 16 selected m^6^A RNA methylation regulators in TCGA ccRCC cohort; **b**, **c** LASSO Cox regression analysis of the selected 5 m^6^A RNA methylation regulators; **d** The survival analysis of the two subgroups stratified based on the median of risk scores calculated by multivariate Cox result; **e** The ROC curve for evaluating the prediction efficiency of the prognostic signature; **f**, **g** The distributions of prognostic signature-based risk scores and its corresponding expression profiles. The red dots represent high-risk patients, green dots represent low-risk patients

The entire group (n = 519) with complete survival information was randomly divided into training group (n = 260) (Additional file 4: Table S1) and validation group (n = 259) (Additional file 5: Table S2) by utilizing “caret” R package. In order to investigate the prognostic role of m^6^A RNA methylation regulators in ccRCC, univariate Cox regression analysis was firstly conducted in training set to identify regulators associated with OS in TCGA ccRCC cohort. The results exhibited that 5 out of 16 regulators were significantly associated with OS (p < 0.05), among which two regulators (HNRNPA2B1 and METTL3) were risky genes with HR > 1 whereas three regulators (METTL14, YTHDC1, YTHDF2) acted as protective genes with HR < 1 (Fig. 4a). In order to better predict the clinical outcomes of ccRCC through m^6^A RNA methylation regulators, these five genes were included in the LASSO Cox regression analysis. Based on the minimum criteria, two genes (METTL3 and METTL14) were screened out (Fig. 4b and c). Importantly, a consistent expression pattern of the two genes were validated both in our own clinical samples (tumor *vs* normal samples, p < 0.05) and in cell lines (human ccRCC cell line *vs* normal kidney cell line, p < 0.05) (Additional file 6: Fig. S4a–d). Subsequently, those two genes were subjected to a stepwise multivariate Cox regression to construct the optimal risk signature (Additional file 7: Table S3). Coefficients generated from multivariate Cox analysis were applied to calculate each ccRCC patient’s risk score using the following formula: risk score = (− 0.385) × METTL14 + (0.121) × METTL3.

In order to evaluate the prognostic role of the two-gene risk signature, the ccRCC patients in training group were divided into high-risk group and low-risk group based on the median risk score calculated above, and the OS between these two groups was compared. The results demonstrated that patients in high-risk group had a significantly worse survival than those in the low risk group (p < 0.001) (Fig. 4d). The ROC curve indicated that the prognostic signature had an acceptable prediction efficiency with the AUC value equal to 0.721 (Fig. 4e). The distributions of two-gene signature-based risk scores and its corresponding expression profiles were displayed in Fig. 4f and g. Taken together, these results indicated that this two-gene risk signature could effectively screen out high-risk ccRCC patients with relatively worse clinical outcome.

### The signature-based risk score was an independent prognostic factor in ccRCC

**Fig. 5:**
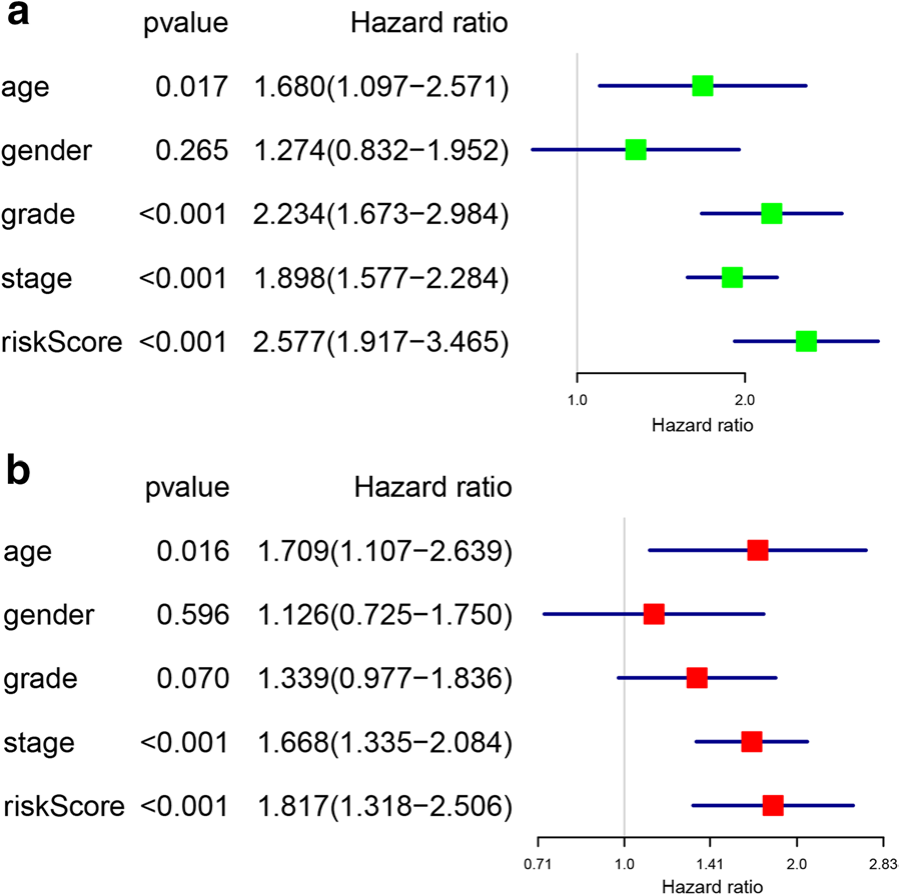
Identification of the independent prognostic factors in the training group. **a** Univariate Cox analyses of the signature based risk score and clinicopathological parameters in training group; **b** Multivariate Cox analyses of the signature based risk score and clinicopathological parameters in training group

In order to determine whether the two-gene risk signature acts as an independent prognostic indicator, we performed univariate and multivariate Cox regression analyses of signature-based risk score in training group. The univariate Cox analysis indicated that signature-based risk score was significantly associated with worse OS with HR = 2.577 (p < 0.001, 95% CI [1.917–3.465]) (Fig. 5a). Meanwhile, age (HR = 1.680, 95% CI [1.097–2.571], p = 0.017), grade (HR = 2.234, 95% CI [1.673–2.984], p < 0.001) and stage (HR = 1.898, 95% CI [1.577–2.284], p < 0.001) were also proved to be significantly associated with the OS (Fig. 5a). After that, all the variables were enlisted into the multivariate Cox analysis. Notably, the signature-based risk score still exerted as risky factor for lower overall survival (HR = 1.817, 95% CI [1.318–2.506], p < 0.001) of ccRCC patients (Fig. 5b). Hence, these data demonstrated that the signature-based risk score was an independent prognostic factor in ccRCC.

**Fig. 6:**
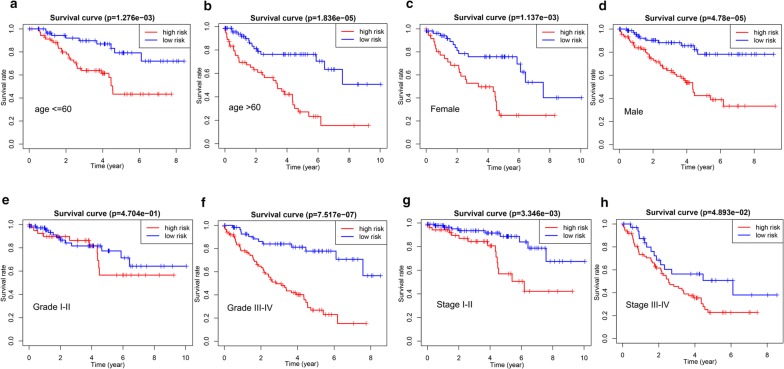
The survival analyses for the low- and high-risk subgroups stratified by clinicopathological parameters in the training group. **a**, **b** The survival analyses for the low- and high-risk subgroups stratified by age; **c**, **d** The survival analyses for the low- and high-risk subgroups stratified by gender; **e**, **f** The survival analyses for the low- and high-risk subgroups stratified by Grade; **g**, **h** The survival analyses for the low- and high-risk subgroups stratified by stage

Subsequently, subgroup analysis was further performed to evaluate the prognostic value of the two-gene risk signature in patients with different clinicopathological features, including age, gender, grade and stage. As shown in Fig. 6a–h, except for patients with grade I–II (p > 0.05), high-risk patients in all subgroup had dramatically lower OS than patients with low-risk (p < 0.05), suggesting that the two-gene risk gene possessed stable discrimination ability for patients with poor prognosis.

### Validation of the prognostic signature

**Fig. 7:**
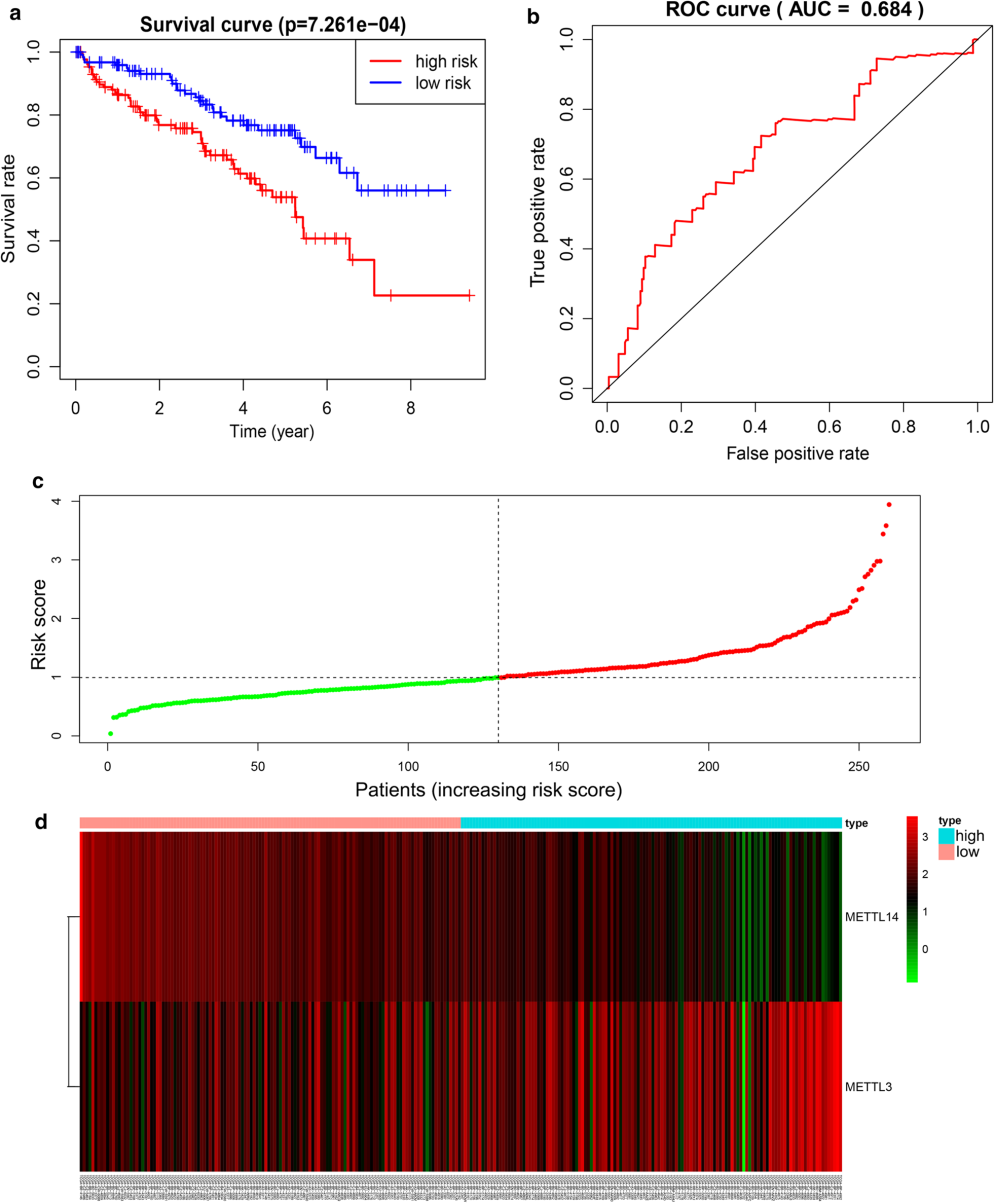
Validation of the prognostic risk signature in the validation group. **a** The survival analysis of the two subgroups stratified based on the median of risk scores calculated by the prognostic risk signature; **b** The ROC curve for evaluating the prediction efficiency of the prognostic signature; **c**, **d** The distributions of prognostic signature-based risk scores and its corresponding expression profiles. The red dots represent high-risk patients, green dots represent low-risk patients

The prognostic value of the two-gene risk signature was validated in TCGA validation cohort (n = 260) and in our own clinical dataset (n = 20). Based on median value of risk score, the Kaplan–Meier curve demonstrated that patients in high risk group had an obviously poorer OS compared to patients with low risk in both of these two validation datasets (p < 0.05) (Fig. 7a and Additional file 8: Fig. S5a). The ROC curves also demonstrated that risk score had a good predictive ability with AUC equal to 0.684 and 0.828, respectively (Fig. 7b and Additional file 8: Fig. S5b). The distributions of the risk scores and expression profiles were shown in Fig. 7c and d. Patients with high risk score had higher mortality rates than low risk patients both in training and validation group (Additional file 9: Fig. S6a and b).

**Fig. 8:**
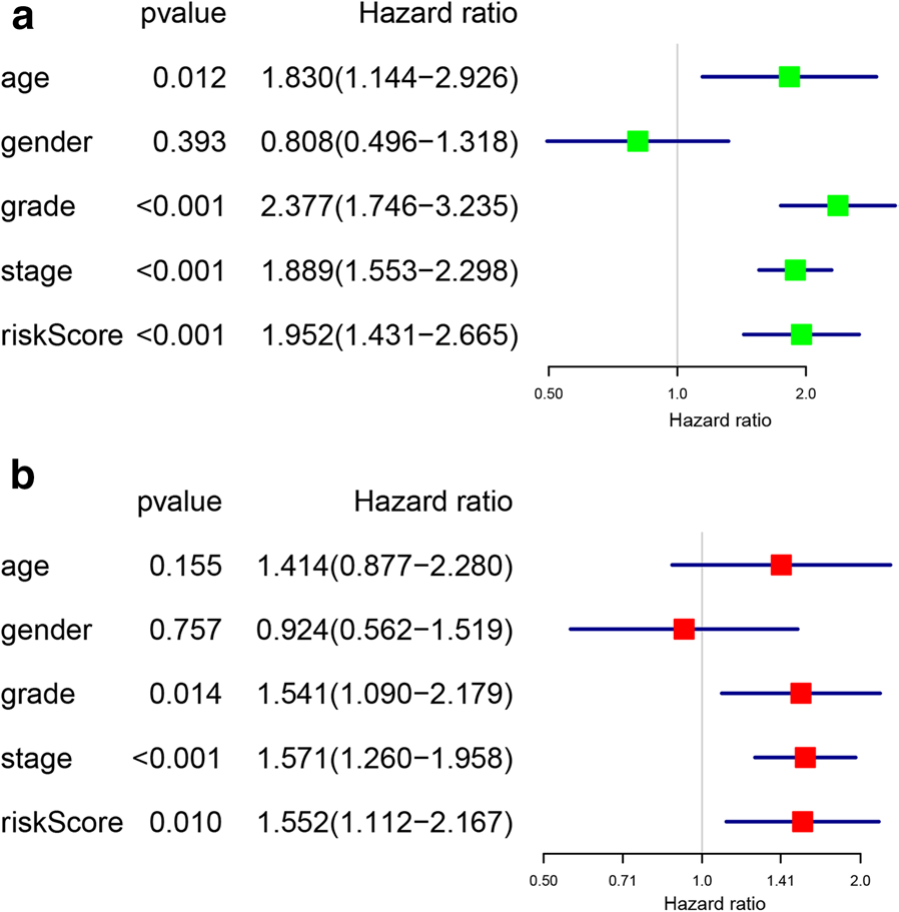
Identification of the independent prognostic factors in the validation group. **a** Univariate Cox analyses of the signature based risk score and clinicopathological parameters in validation group; **b** Multivariate Cox analyses of the signature based risk score and clinicopathological parameters in validation group

**Fig. 9:**
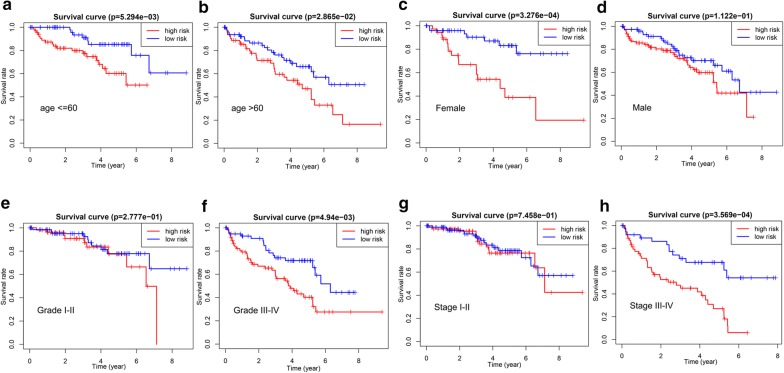
The survival analyses for the low- and high-risk subgroups stratified by clinicopathological parameters in the validation group. **a**, **b** The survival analyses for the low- and high-risk subgroups stratified by age; **c**, **d** The survival analyses for the low- and high-risk subgroups stratified by gender; **e**, **f** The survival analyses for the low- and high-risk subgroups stratified by grade; **g**, **h** The survival analyses for the low- and high-risk subgroups stratified by stage

Consistent with the training set, univariate analysis demonstrated that age (HR = 1.830, 95% CI [1.144–2.926], p = 0.012), grade (HR = 2.377, 95% CI [1.746–3.235], p < 0.001), stage (HR = 1.889, 95% CI [1.553–2.298], p < 0.001) and signature-based risk score (HR = 1.952, 95% CI [1.431–2.665], p < 0.001) were significantly associated with the OS in validation set (Fig. 8a). The multivariate analysis further showed that signature-based risk score served as independent prognostic indicators (HR = 1.552, 95% CI [1.112–2.167], p = 0.010) (Fig. 8b). Moreover, subgroup analysis in the validation group revealed similar results that high-risk patients had an obviously poorer OS compared to low-risk patients except for patients with early stage (stage I–II, p < 0.05) or low grade (grade I–II, p < 0.05) (Fig. 9a–h). These results, taken together, convincingly verified the prognostic value of this two-gene risk signature in ccRCC patients.

## Discussion

Recently, m^6^A RNA modifications acted by methyltransferases, demethylases and binding proteins are demonstrated to be critical regulators of mRNA stability, splicing, processing and translation [22–24]. By means of the opposing m^6^A methylation and demethylation mechanisms, emerging evidences have indicated that m^6^A RNA modifications can mediate various malignancy-related process, such as onco-transcript expression, tumorigenesis [25], tumor proliferation [26], invasion [25], and metastasis [27]. On the contrary, anti-oncogenic role of m^6^A methylation have also been reported by various studies. For instance, Liu et al. reported that reduced m^6^A methylation regulates AKT activity to promote the proliferation and tumorigenicity of endometrial cancer, implying its tumor suppressive role especially in endometrial cancer [28]. Moreover, m^6^A modifications were found to be decreased in hepatocellular carcinoma and involved in suppressing the tumor metastasis [29]. Given that m^6^A modifications is a double-edged sword which plays a dual role of either as a tumor suppressor or an oncogene in multiple tumors, its exact role in other cancers, such as ccRCC, needs to be further elucidated.

In this present study, we proved that 12 out of 16 m^6^A RNA methylation regulators were abnormally expressed in ccRCC. Interestingly, multiple genes were relatively downregulated in ccRCC and negatively correlated with the malignancy of the tumor. This is not consistent with previous studies focusing on gliomas and gastric carcinoma wherein the majority of m^6^A RNA methylation regulators were highly expressed in tumor tissue [30, 31], implying its distinct role in ccRCC. In addition, two clusters of ccRCC subgroups were identified based on the expression pattern of m^6^A RNA methylation regulators by means of consensus clustering. The results showed a significant difference in OS and tumor stage between the two subgroups, which further implied that the expression pattern of m^6^A related genes were closely related to the malignancy and prognosis of ccRCC. Previous studies reported that RNA m^6^A methylation could affect various biological processes and signaling pathways, such as self-renewal and tumorigenesis of cancer stem cells [32], RNA metabolism [33], the FTO/m6A/MYC/CEBPA signaling [34], and the IL-7/STAT5/SOCS pathways [35]. In our study, similarly, the functional analysis of the differentially regulated genes between the two clusters of ccRCC showed significantly associated with malignancy-related biological processes as well as signaling pathways. These results, taken together, uncovered that RNA m^6^A methylation could play a vital role in regulating the malignant process of ccRCC.

Previous study indicated that ccRCC patients with any copy number variations (CNVs) of the m^6^A RNA methylation regulators had worse OS and DFS than those with diploid genes [36]. In our study, more interestingly, we performed univariate, LASSO, and multivariate Cox regression analyses to develop a prognostic related risk signature with two m^6^A RNA methylation regulators, METTL3 and METTL14, which divided the ccRCC patients into low- and high-risk groups. As a result, the two-gene signature significantly distinguished patients with different OS (p < 0.05), and showed a good performance for predicting patients’ prognosis. Moreover, the following multivariate Cox analysis further demonstrated this two-gene risk signature as an independent prognostic marker with a higher HR value than other clinicopathological factor including age, grade and stage. More importantly, a dramatical difference of OS between low- and high risk groups was also observed in ccRCC patients stratified by some clinicopathological features. Although no significant OS difference was found in subgroup of grade I-II patients, we observed that survival rate of patients with high-risk were relatively lower than those with low-risk after 4 years of follow-up. We speculated that this might be due to the limited number of patients in TCGA cohort. Finally, we validated this two-gene risk signature in validation group, which further suggested its convincing prognostic value in ccRCC patients.

Our prognostic two-gene signature showed that METTL3 was a risky gene for the prognosis of ccRCC while METTL14 acted as a protective gene. It is well demonstrated that METTL14 can form stable complexes with METTL13 [37], and function as a pseudo- methyltransferase that stabilizes METTL3 and recognizes target RNA [38]. Interestingly, these two “writer” genes, METTL3 and METTL14, showed an opposite effect on survival of ccRCC patients, which hinted that the “writers” exerted as a complicated regulators in ccRCC. Currently, the roles of “writer” genes METTL3 and METTL14 have been explored by various studies. For example, METTL3 was reported to act as an oncogene associated with the tumor progression and metastasis in various cancers, such as gastric cancer [18], colorectal cancer [27], and pancreatic cancer [39]. On the contrary, other studies also demonstrated that METTL3 played an suppressive role in endometrial cancer and glioblastoma [28, 32]. Similarly, emerging evidences have also indicated that METTL14 could exert as either a oncogene or tumor suppressor [29, 40]. Considering the opposite effect of the two “writer” genes in various cancers or even in the same cancer type, more experimental proofs regarding to the exact role of METTL3 and METTL14 in ccRCC are in high demand in future.

## Conclusions

In summary, our study systematically demonstrated a dysregulated expression of m^6^A RNA methylation regulators between ccRCC and normal controls. The m^6^A RNA methylation regulators were also significantly associated with the clinicopathological features, which implied its crucial role in the tumorigenesis and progression of ccRCC. In addition, a two-gene risky signature was successfully constructed to distinguish ccRCC patients with different prognosis, indicating its prognostic value as a promising molecular biomarker.

## Supplementary information

**Additional file 1: Figure S1.** Identification of hub genes in selected 16 RNA methylation regulators.

**Additional file 2: Figure S2.** Identification of consensus clusters by m^6^A RNA methylation regulators. (a) Consensus clustering matrix for k = 2; (b) The tracking plot for k = 2 to 10.

**Additional file 3: Figure S3.** Functional analysis for the differentially expressed mRNAs between cluster 1 and cluster 2 subgroups. (a) Enriched BP items; (b) Enriched KEGG pathways.

**Additional file 4: Table S1.** Patients information in training group.

**Additional file 5: Table S2.** Patients information in validation group.

**Additional file 6: Figure S4.** Validation of METTL3 and METTL14 through qRT-PCR. Expression of METTL3 (a) and METTL14 (b) in human ccRCC clinical samples compared with normal kidney samples; Expression of METTL3 (c) and METTL14 (d) in human ccRCC cell line (786-O) compared with normal proximal tubule epithelial cell line (HK2). *P < 0.05, **P < 0.01.

**Additional file 7: Table S3.** Coefficients based on a multivariate Cox regression analysis.

**Additional file 8: Figure S5.** Validation of the prognostic risk signature in our own clinical dataset. (a) The survival analysis of the two subgroups stratified based on the median of risk scores calculated by the prognostic risk signature; (b) The ROC curve for evaluating the prediction efficiency of the prognostic signature.

**Additional file 9: Figure S6.** Survival status in high and low risk patients for training group (a) and validation group (b). red dots represent death, green dots represent alive.

## Data Availability

All data are available from the sources listed in the manuscript—the TCGA data portal.

## Abbreviations

ccRCC: Clear cell renal cell carcinoma
TCGA: The Cancer Genome Atlas
RCC: Renal cell carcinoma
LASSO: Least absolute shrinkage and selection operator
OS: Overall survival
RFS: Recurrence-free survival
lncRNAs: Long non-coding RNAs
GO: Gene ontology
KEGG: Kyoto Encyclopedia of Genes and Genomes
DEGs: Differentially expressed genes
DAVID: Database of Annotation, Visualization and Integrated Discovery
PPI: Protein–protein interaction
ROC: Receiver operating characteristic
