# Supplementary figures and images for: Identification of a m6A RNA methylation regulators-based signature for predicting the prognosis of clear cell renal carcinoma
Source: Cancer Cell Int. 2020 May 7;20:157. doi: 10.1186/s12935-020-01238-3 (PMC7206820; doi:10.1186/s12935-020-01238-3)

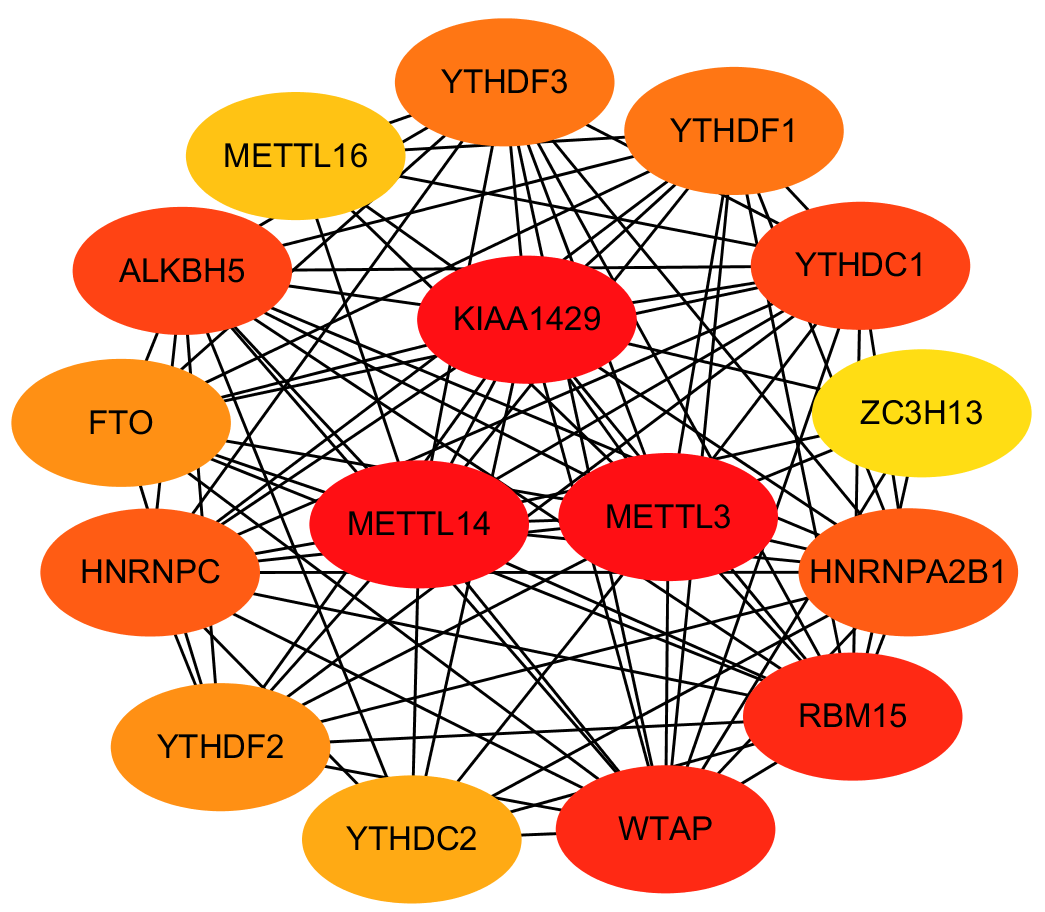

Supplement: Supplementary file 1 — Additional file 1: Figure S1. Identification of hub genes in selected 16 RNA methylation regulators. [file 12935_2020_1238_MOESM1_ESM.tif]

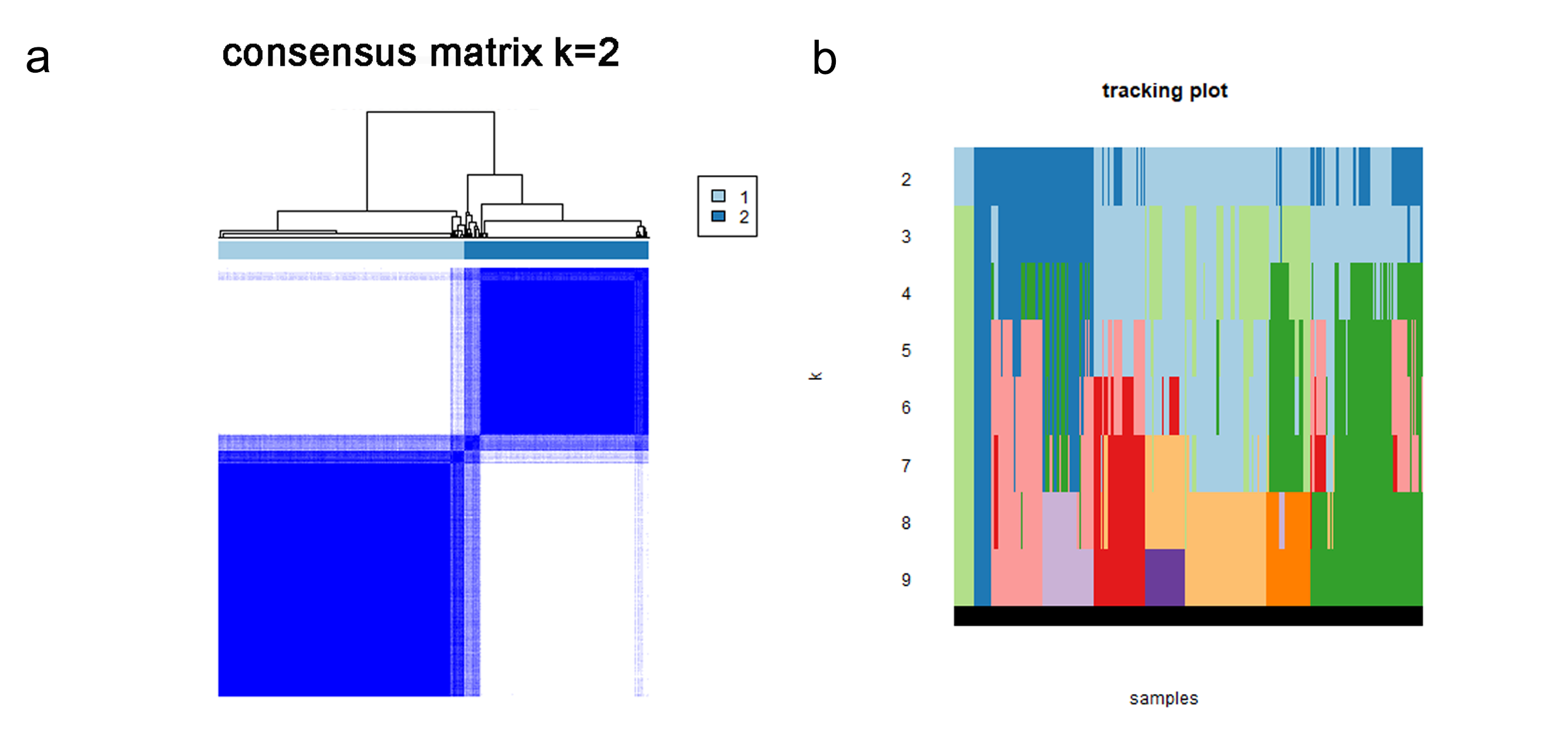

Supplement: Supplementary file 2 — Additional file 2: Figure S2. Identification of consensus clusters by m6A RNA methylation regulators. (a) Consensus clustering matrix for k = 2; (b) The tracking plot for k = 2 to 10. [file 12935_2020_1238_MOESM2_ESM.tif]

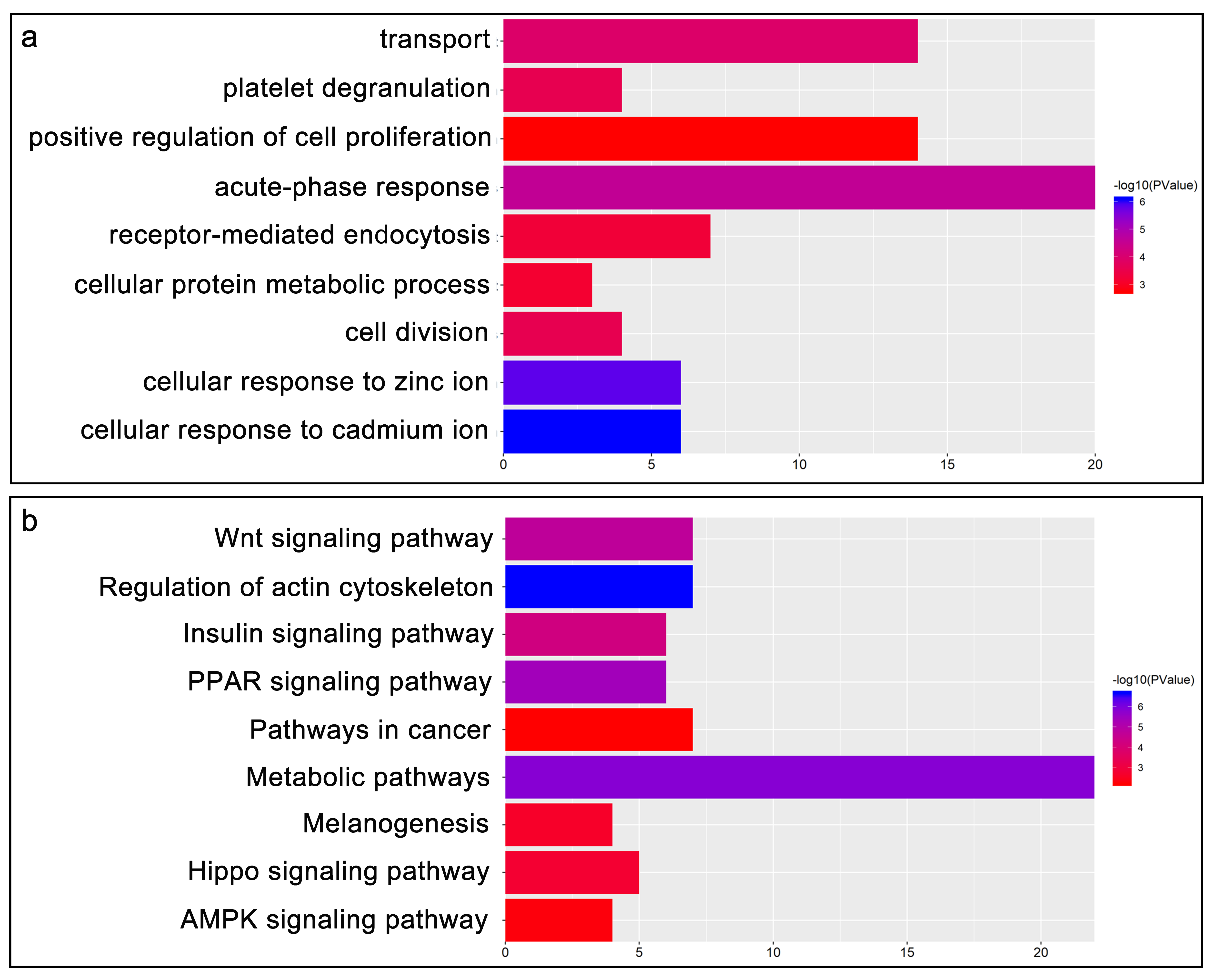

Supplement: Supplementary file 3 — Additional file 3: Figure S3. Functional analysis for the differentially expressed mRNAs between cluster 1 and cluster 2 subgroups. (a) Enriched BP items; (b) Enriched KEGG pathways. [file 12935_2020_1238_MOESM3_ESM.tif]

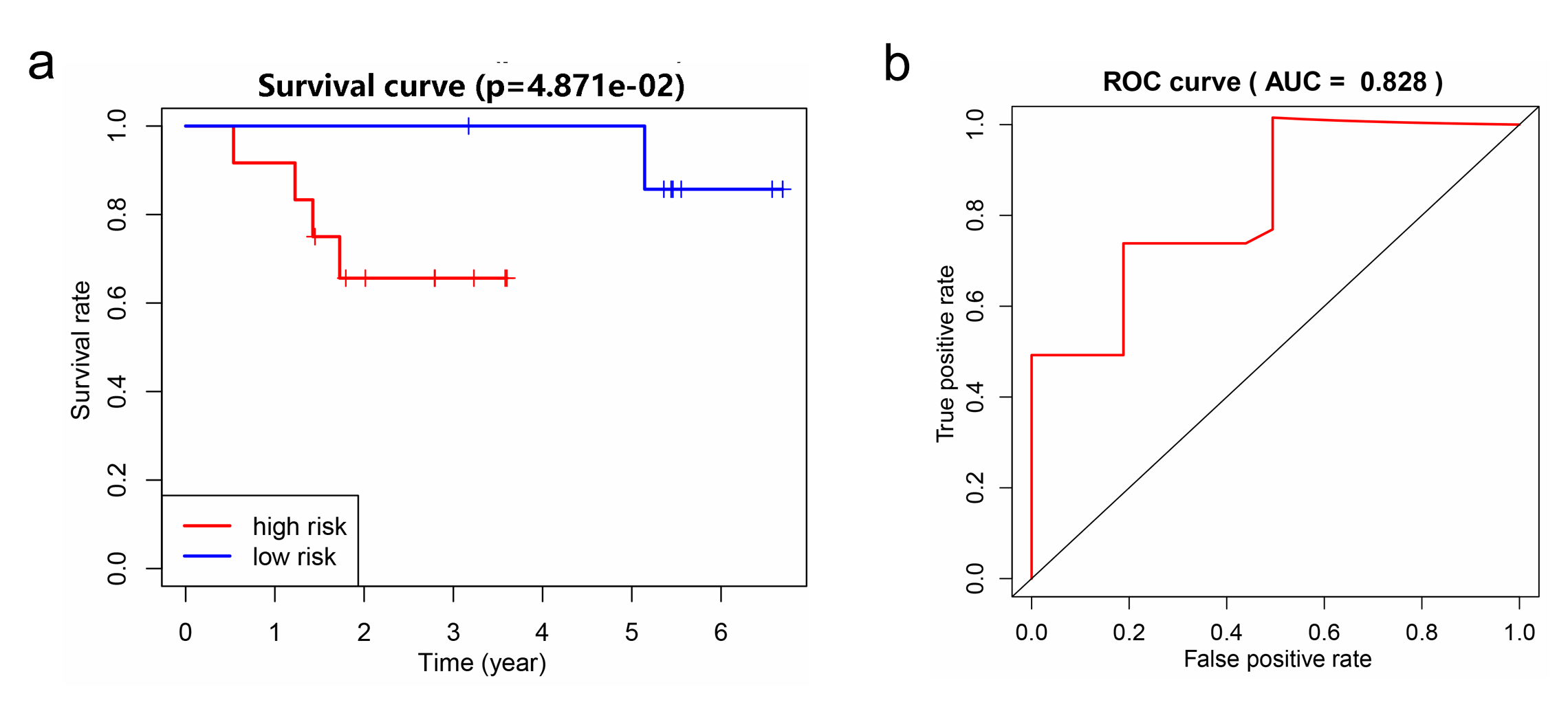

Supplement: Supplementary file 6 — Additional file 6: Figure S4. Validation of METTL3 and METTL14 through qRT-PCR. Expression of METTL3 (a) and METTL14 (b) in human ccRCC clinical samples compared with normal kidney samples; Expression of METTL3 (c) and METTL14 (d) in human ccRCC cell line (786-O) compared with normal proximal tubule epithelial cell line (HK2). *P < 0.05, **P < 0.01. [file 12935_2020_1238_MOESM6_ESM.tif]

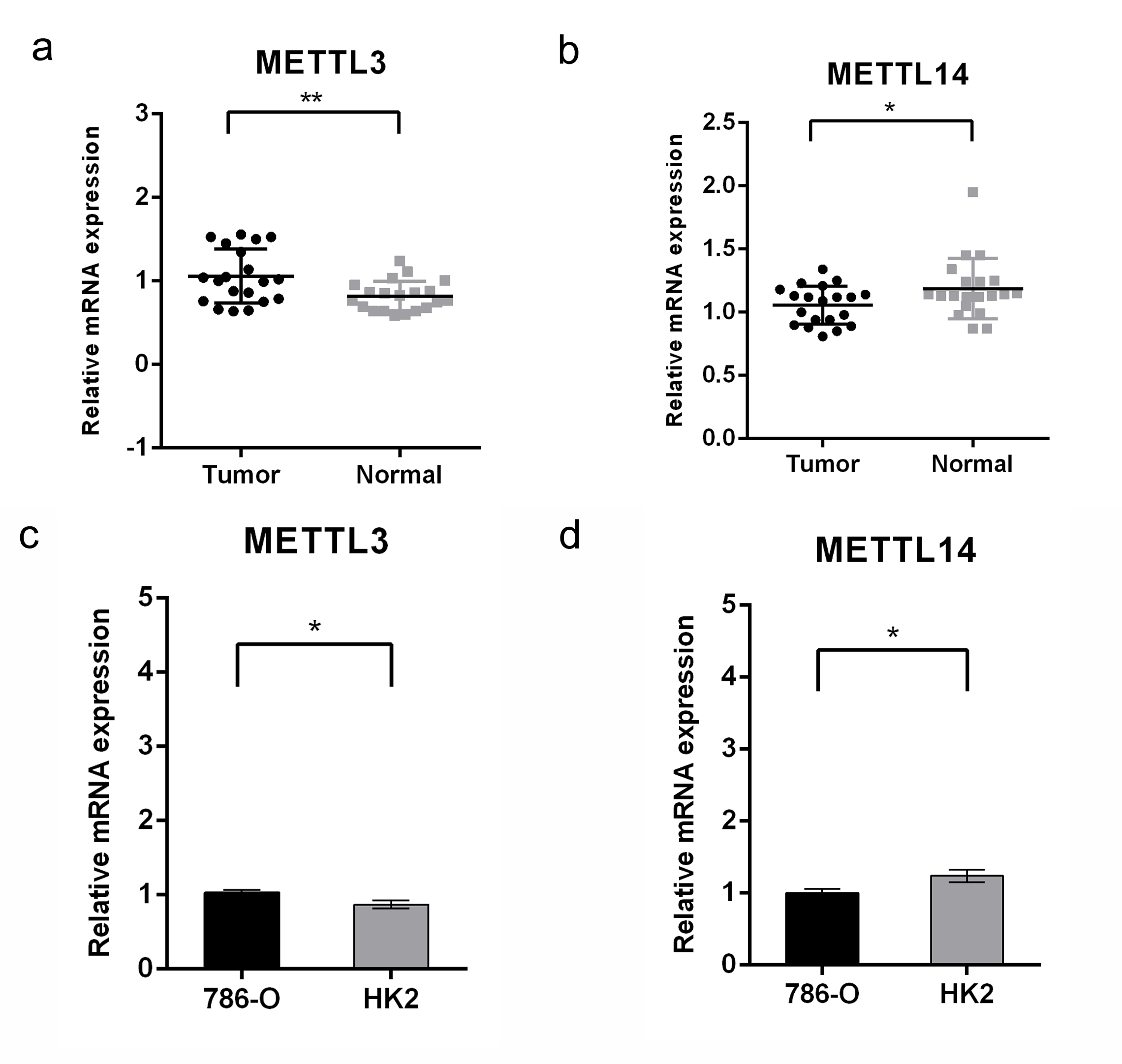

Supplement: Supplementary file 8 — Additional file 8: Figure S5. Validation of the prognostic risk signature in our own clinical dataset. (a) The survival analysis of the two subgroups stratified based on the median of risk scores calculated by the prognostic risk signature; (b) The ROC curve for evaluating the prediction efficiency of the prognostic signature. [file 12935_2020_1238_MOESM8_ESM.tif]

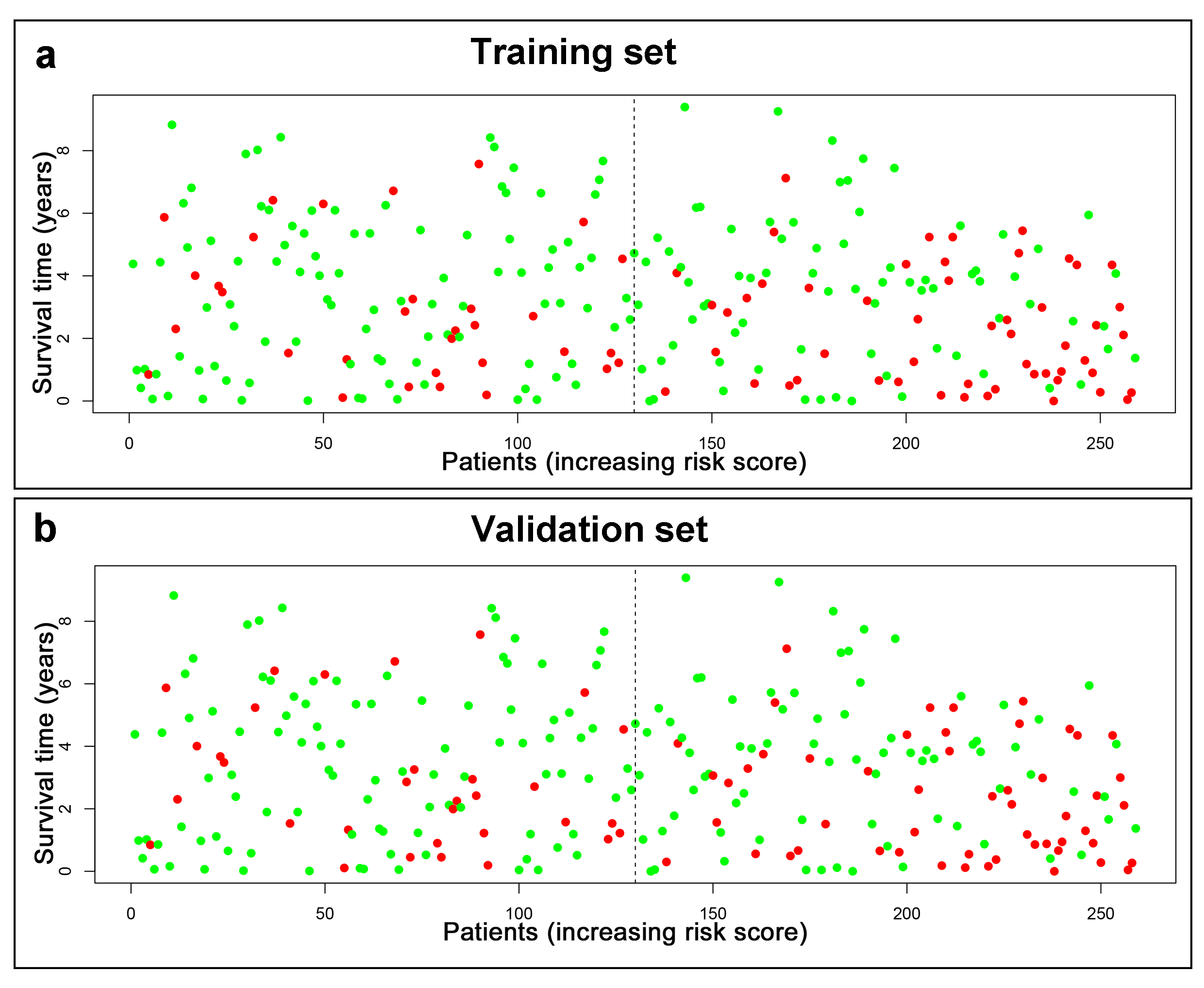

Supplement: Supplementary file 9 — Additional file 9: Figure S6. Survival status in high and low risk patients for training group (a) and validation group (b). red dots represent death, green dots represent alive. [file 12935_2020_1238_MOESM9_ESM.tif]
