# Supplementary material for: Identification of a m6A RNA methylation regulators-based signature for predicting the prognosis of clear cell renal carcinoma
Source: Cancer Cell Int. 2020 May 7;20:157. doi: 10.1186/s12935-020-01238-3 (PMC7206820; doi:10.1186/s12935-020-01238-3)
Supplement: Supplementary file 4 — Additional file 4: Table S1. Patients information in training group. [file 12935_2020_1238_MOESM4_ESM.docx]

Table S1. Patients information in training group.

| id | survival time (year) | survival status |
| --- | --- | --- |
| TCGA-CJ-4882 | 5.158904 | 0 |
| TCGA-DV-A4W0 | 5.50137 | 0 |
| TCGA-CJ-6030 | 6.29863 | 1 |
| TCGA-CJ-6032 | 6.980822 | 0 |
| TCGA-B0-5100 | 5.241096 | 1 |
| TCGA-A3-3352 | 1.536986 | 1 |
| TCGA-BP-5173 | 0.169863 | 1 |
| TCGA-CZ-4859 | 4.89589 | 0 |
| TCGA-BP-5199 | 3.712329 | 0 |
| TCGA-A3-3387 | 1.690411 | 0 |
| TCGA-BP-5198 | 1.652055 | 0 |
| TCGA-CZ-5468 | 0.161644 | 1 |
| TCGA-BP-4161 | 7.523288 | 0 |
| TCGA-BP-5175 | 2.553425 | 0 |
| TCGA-DV-A4VZ | 1 | 0 |
| TCGA-A3-3378 | 1.726027 | 0 |
| TCGA-BP-5189 | 2.252055 | 1 |
| TCGA-BP-5006 | 2.30137 | 0 |
| TCGA-BP-5178 | 5.238356 | 1 |
| TCGA-B0-4852 | 3.071233 | 1 |
| TCGA-CZ-5456 | 4.268493 | 0 |
| TCGA-CZ-5986 | 1.021918 | 0 |
| TCGA-BP-4346 | 4.090411 | 1 |
| TCGA-B0-5694 | 1.315068 | 1 |
| TCGA-CJ-4643 | 4.912329 | 0 |
| TCGA-DV-5568 | 1.013699 | 0 |
| TCGA-BP-4347 | 3.745205 | 0 |
| TCGA-BP-4790 | 3.043836 | 1 |
| TCGA-T7-A92I | 0.975342 | 0 |
| TCGA-EU-5904 | 1.509589 | 0 |
| TCGA-CJ-5684 | 6.112329 | 0 |
| TCGA-A3-3346 | 0.375342 | 1 |
| TCGA-B0-5110 | 2.991781 | 0 |
| TCGA-B0-4845 | 5.441096 | 1 |
| TCGA-B0-4706 | 0.178082 | 1 |
| TCGA-CW-5583 | 6.819178 | 0 |
| TCGA-B0-5702 | 4.39726 | 0 |
| TCGA-AK-3456 | 3.131507 | 0 |
| TCGA-A3-3311 | 3.263014 | 1 |
| TCGA-B0-5710 | 3.99726 | 0 |
| TCGA-B8-5162 | 0.060274 | 0 |
| TCGA-A3-3370 | 6.230137 | 0 |
| TCGA-CJ-4894 | 2.30411 | 1 |
| TCGA-CJ-6031 | 5.221918 | 0 |
| TCGA-BP-4774 | 5.164384 | 0 |
| TCGA-CW-5588 | 5.526027 | 0 |
| TCGA-BP-4335 | 1.30137 | 1 |
| TCGA-BP-4981 | 3.005479 | 1 |
| TCGA-A3-3362 | 4.271233 | 0 |
| TCGA-A3-3329 | 4.449315 | 0 |
| TCGA-B8-A8YJ | 1.180822 | 0 |
| TCGA-B0-5117 | 1.465753 | 0 |
| TCGA-CZ-5452 | 4.263014 | 0 |
| TCGA-B0-5712 | 7.457534 | 0 |
| TCGA-EU-5905 | 0.326027 | 0 |
| TCGA-CJ-4903 | 4.273973 | 0 |
| TCGA-B8-4621 | 1.180822 | 0 |
| TCGA-A3-3323 | 3.030137 | 0 |
| TCGA-B8-5164 | 0.063014 | 0 |
| TCGA-BP-4975 | 3.926027 | 0 |
| TCGA-B8-4143 | 1.942466 | 1 |
| TCGA-BP-5168 | 4.008219 | 1 |
| TCGA-B4-5377 | 1 | 0 |
| TCGA-MM-A84U | 1.917808 | 0 |
| TCGA-B8-5545 | 1.430137 | 0 |
| TCGA-BP-4985 | 2.608219 | 1 |
| TCGA-BP-4159 | 7.126027 | 1 |
| TCGA-B2-5641 | 0.887671 | 0 |
| TCGA-BP-5190 | 2.769863 | 0 |
| TCGA-B0-5697 | 5.027397 | 0 |
| TCGA-B0-5706 | 6.613699 | 0 |
| TCGA-BP-5001 | 3.224658 | 0 |
| TCGA-AK-3429 | 5.526027 | 0 |
| TCGA-AK-3447 | 3.334247 | 0 |
| TCGA-BP-4351 | 2.657534 | 0 |
| TCGA-BP-4331 | 6.723288 | 1 |
| TCGA-AK-3445 | 3.506849 | 0 |
| TCGA-CW-6088 | 8.827397 | 0 |
| TCGA-A3-3347 | 4.410959 | 1 |
| TCGA-CJ-4888 | 4.293151 | 1 |
| TCGA-BP-4160 | 7.893151 | 0 |
| TCGA-BP-4765 | 5.983562 | 0 |
| TCGA-AS-3778 | 0.117808 | 0 |
| TCGA-A3-3365 | 2.391781 | 0 |
| TCGA-CJ-4893 | 2.054795 | 0 |
| TCGA-CZ-5454 | 1.978082 | 1 |
| TCGA-B0-5113 | 0.983562 | 0 |
| TCGA-BP-5170 | 6.608219 | 0 |
| TCGA-A3-3380 | 1.553425 | 0 |
| TCGA-BP-4756 | 1.024658 | 0 |
| TCGA-BP-4795 | 1.69863 | 0 |
| TCGA-BP-4987 | 3.079452 | 0 |
| TCGA-B0-4691 | 0.380822 | 1 |
| TCGA-B8-4622 | 0.49589 | 0 |
| TCGA-B0-4816 | 3.756164 | 1 |
| TCGA-CZ-5455 | 1.536986 | 1 |
| TCGA-BP-5196 | 2.789041 | 0 |
| TCGA-B8-5551 | 0.041096 | 0 |
| TCGA-A3-3331 | 3.443836 | 0 |
| TCGA-B0-4811 | 3.882192 | 1 |
| TCGA-B0-5400 | 3.10137 | 0 |
| TCGA-BP-5182 | 3.191781 | 0 |
| TCGA-A3-3335 | 5.167123 | 0 |
| TCGA-B0-4718 | 1.687671 | 0 |
| TCGA-BP-4960 | 5.950685 | 0 |
| TCGA-B0-4844 | 0.857534 | 1 |
| TCGA-B2-4099 | 1.024658 | 0 |
| TCGA-AK-3460 | 2.605479 | 0 |
| TCGA-BP-4807 | 0.578082 | 0 |
| TCGA-B0-4833 | 6.536986 | 1 |
| TCGA-A3-3343 | 2.589041 | 0 |
| TCGA-BP-5201 | 2.605479 | 0 |
| TCGA-BP-4969 | 4.915068 | 0 |
| TCGA-A3-3385 | 5.460274 | 0 |
| TCGA-CJ-4640 | 5.473973 | 0 |
| TCGA-BP-4352 | 0.942466 | 1 |
| TCGA-A3-A6NL | 1.887671 | 0 |
| TCGA-CZ-5459 | 4.150685 | 0 |
| TCGA-B8-4154 | 0.69863 | 0 |
| TCGA-BP-4989 | 0.323288 | 0 |
| TCGA-CJ-4872 | 3.931507 | 0 |
| TCGA-CZ-5463 | 1.813699 | 0 |
| TCGA-CJ-4920 | 0.380822 | 1 |
| TCGA-BP-5009 | 2.991781 | 1 |
| TCGA-BP-4999 | 3.468493 | 0 |
| TCGA-BP-4330 | 5.172603 | 0 |
| TCGA-CZ-4861 | 1.221918 | 1 |
| TCGA-CJ-4912 | 4.539726 | 0 |
| TCGA-B0-4838 | 2.284932 | 1 |
| TCGA-B8-5163 | 0.010959 | 0 |
| TCGA-B0-4814 | 0.460274 | 1 |
| TCGA-BP-4766 | 4.005479 | 0 |
| TCGA-A3-3357 | 7.364384 | 0 |
| TCGA-BP-4962 | 4.890411 | 0 |
| TCGA-B0-5077 | 3.608219 | 1 |
| TCGA-CJ-5681 | 1.512329 | 1 |
| TCGA-BP-5191 | 2.649315 | 0 |
| TCGA-BP-5008 | 2.934247 | 0 |
| TCGA-AK-3428 | 6.090411 | 0 |
| TCGA-G6-A8L8 | 2.989041 | 1 |
| TCGA-CZ-5467 | 0.2 | 1 |
| TCGA-CJ-4634 | 4.986301 | 0 |
| TCGA-B0-5083 | 2.863014 | 1 |
| TCGA-CJ-4871 | 6.638356 | 0 |
| TCGA-CJ-4892 | 4.167123 | 0 |
| TCGA-B8-A54I | 0.410959 | 0 |
| TCGA-B0-5696 | 4.731507 | 0 |
| TCGA-B0-4688 | 0.276712 | 1 |
| TCGA-AK-3450 | 4.131507 | 0 |
| TCGA-B0-5092 | 1.257534 | 1 |
| TCGA-B8-A7U6 | 1.356164 | 0 |
| TCGA-B0-4822 | 3.043836 | 1 |
| TCGA-CJ-4907 | 4.106849 | 0 |
| TCGA-G6-A5PC | 0.663014 | 1 |
| TCGA-B8-4619 | 0.052055 | 0 |
| TCGA-B0-5108 | 2.49589 | 0 |
| TCGA-DV-5567 | 2.493151 | 0 |
| TCGA-BP-5174 | 6.183562 | 0 |
| TCGA-BP-4176 | 5.356164 | 0 |
| TCGA-BP-4349 | 1.019178 | 0 |
| TCGA-B0-4699 | 0.30137 | 1 |
| TCGA-CJ-4642 | 4.460274 | 0 |
| TCGA-BP-4343 | 5.238356 | 1 |
| TCGA-B8-A54D | 2.273973 | 0 |
| TCGA-BP-4787 | 1.315068 | 1 |
| TCGA-B0-4703 | 0.49863 | 1 |
| TCGA-DV-5573 | 3.09589 | 0 |
| TCGA-B0-4846 | 3.287671 | 1 |
| TCGA-A3-3308 | 0.043836 | 0 |
| TCGA-B8-4620 | 0.619178 | 0 |
| TCGA-B0-4834 | 5.726027 | 1 |
| TCGA-B2-5633 | 0.980822 | 0 |
| TCGA-CJ-4636 | 5.271233 | 0 |
| TCGA-BP-4797 | 3.032877 | 0 |
| TCGA-CJ-4886 | 5.347945 | 0 |
| TCGA-B0-4700 | 5.424658 | 1 |
| TCGA-A3-3307 | 3.934247 | 0 |
| TCGA-B0-5713 | 5.109589 | 0 |
| TCGA-CJ-4908 | 4.194521 | 0 |
| TCGA-BP-4967 | 0.561644 | 0 |
| TCGA-CJ-4916 | 3.761644 | 0 |
| TCGA-B0-4848 | 2.419178 | 1 |
| TCGA-CJ-4875 | 6.446575 | 0 |
| TCGA-DV-5575 | 2.756164 | 0 |
| TCGA-CZ-5451 | 4.569863 | 0 |
| TCGA-A3-3351 | 2.493151 | 0 |
| TCGA-B8-4146 | 1.4 | 0 |
| TCGA-CW-6087 | 0.112329 | 1 |
| TCGA-B4-5834 | 0.10411 | 0 |
| TCGA-B0-4693 | 0.210959 | 1 |
| TCGA-B0-5080 | 0.936986 | 1 |
| TCGA-AK-3454 | 2.394521 | 0 |
| TCGA-B0-4837 | 3.775342 | 1 |
| TCGA-BP-5187 | 1.112329 | 0 |
| TCGA-A3-3349 | 3.794521 | 0 |
| TCGA-CJ-6027 | 5.082192 | 0 |
| TCGA-BP-5000 | 1.542466 | 0 |
| TCGA-CW-5581 | 7.668493 | 0 |
| TCGA-CW-5590 | 2.945205 | 1 |
| TCGA-B0-5109 | 1.608219 | 1 |
| TCGA-BP-4325 | 8.120548 | 0 |
| TCGA-A3-3326 | 3.115068 | 0 |
| TCGA-CZ-4863 | 4.632877 | 0 |
| TCGA-BP-4771 | 0.443836 | 1 |
| TCGA-B0-4827 | 2.424658 | 1 |
| TCGA-MM-A564 | 1.663014 | 0 |
| TCGA-B0-5700 | 2.964384 | 0 |
| TCGA-A3-A8OW | 0.884932 | 0 |
| TCGA-B4-5835 | 0.043836 | 0 |
| TCGA-BP-4169 | 1.920548 | 1 |
| TCGA-BP-4345 | 4.153425 | 0 |
| TCGA-B0-5691 | 9.4 | 0 |
| TCGA-B8-A54H | 0.70137 | 0 |
| TCGA-B0-4714 | 0.271233 | 1 |
| TCGA-AK-3434 | 5.717808 | 0 |
| TCGA-B0-4849 | 0.189041 | 1 |
| TCGA-A3-3374 | 3.6 | 0 |
| TCGA-BP-4332 | 3.10411 | 0 |
| TCGA-BP-4763 | 3.479452 | 1 |
| TCGA-BP-4162 | 8.421918 | 0 |
| TCGA-A3-A6NJ | 1.282192 | 0 |
| TCGA-B0-5701 | 4.745205 | 0 |
| TCGA-B8-A54F | 1.421918 | 0 |
| TCGA-BP-5186 | 1.89863 | 0 |
| TCGA-A3-3373 | 4.441096 | 0 |
| TCGA-A3-3367 | 6.219178 | 0 |
| TCGA-6D-AA2E | 0.991781 | 0 |
| TCGA-BP-4968 | 4.783562 | 0 |
| TCGA-BP-4760 | 6.468493 | 0 |
| TCGA-B0-5692 | 4.073973 | 0 |
| TCGA-CW-5591 | 6.221918 | 0 |
| TCGA-DV-5574 | 1.980822 | 0 |
| TCGA-BP-4963 | 5.024658 | 0 |
| TCGA-BP-4992 | 1.372603 | 0 |
| TCGA-BP-4769 | 5.139726 | 0 |
| TCGA-BP-5183 | 3.536986 | 0 |
| TCGA-CZ-4864 | 3.60274 | 1 |
| TCGA-BP-4777 | 4.742466 | 0 |
| TCGA-BP-4961 | 5.30137 | 0 |
| TCGA-AK-3431 | 5.076712 | 0 |
| TCGA-CZ-5982 | 5.594521 | 0 |
| TCGA-B0-4696 | 2.372603 | 1 |
| TCGA-AK-3440 | 4.780822 | 0 |
| TCGA-A3-3325 | 3.205479 | 1 |
| TCGA-A3-A8CQ | 0.008219 | 0 |
| TCGA-CW-5580 | 5.380822 | 1 |
| TCGA-B8-4148 | 1.038356 | 0 |
| TCGA-B0-4817 | 2.791781 | 1 |
| TCGA-EU-5906 | 0.564384 | 0 |
| TCGA-BP-4163 | 7.778082 | 0 |
| TCGA-CZ-5453 | 0.068493 | 0 |
| TCGA-CJ-4899 | 4.186301 | 0 |
| TCGA-B0-5703 | 3.29589 | 0 |
| TCGA-B0-5081 | 0.991781 | 1 |
| TCGA-A3-3317 | 4.084932 | 0 |
| TCGA-B0-4823 | 1.243836 | 1 |
| TCGA-CZ-4860 | 0.564384 | 1 |
| TCGA-A3-A8OV | 0.931507 | 0 |
| TCGA-BP-5176 | 4.356164 | 1 |
| TCGA-CJ-4900 | 4.69589 | 1 |

Survival status: 0 represent alive, 1represent dead.
