# Supplementary material for: Identification of a m6A RNA methylation regulators-based signature for predicting the prognosis of clear cell renal carcinoma
Source: Cancer Cell Int. 2020 May 7;20:157. doi: 10.1186/s12935-020-01238-3 (PMC7206820; doi:10.1186/s12935-020-01238-3)
Supplement: Supplementary file 5 — Additional file 5: Table S2. Patients information in validation group. [file 12935_2020_1238_MOESM5_ESM.docx]

Table S1. Patients information in validation group.

| id | Survival time (year) | Survival status |
| --- | --- | --- |
| TCGA-BP-4166 | 0.035616 | 0 |
| TCGA-BP-4995 | 3.756164 | 0 |
| TCGA-B0-5699 | 7.509589 | 0 |
| TCGA-A3-A6NI | 2.789041 | 0 |
| TCGA-CJ-4873 | 4.865753 | 0 |
| TCGA-CJ-5676 | 7.054795 | 0 |
| TCGA-A3-3383 | 2.358904 | 0 |
| TCGA-BP-4804 | 3.99726 | 0 |
| TCGA-CZ-5464 | 4.087671 | 0 |
| TCGA-BP-4173 | 5.186301 | 0 |
| TCGA-AK-3458 | 3.2 | 0 |
| TCGA-CJ-4635 | 3.879452 | 0 |
| TCGA-BP-4973 | 3.791781 | 0 |
| TCGA-B0-4828 | 0.841096 | 1 |
| TCGA-B0-4945 | 5.876712 | 1 |
| TCGA-B8-5158 | 0.80274 | 0 |
| TCGA-CJ-5679 | 1.860274 | 1 |
| TCGA-BP-5007 | 3.123288 | 0 |
| TCGA-BP-4340 | 1.539726 | 1 |
| TCGA-CJ-4876 | 5.356164 | 0 |
| TCGA-B8-5546 | 1.383562 | 0 |
| TCGA-A3-A8OX | 0 | 0 |
| TCGA-B0-5102 | 7.572603 | 1 |
| TCGA-B0-4701 | 0.652055 | 1 |
| TCGA-CJ-6033 | 0.613699 | 1 |
| TCGA-BP-5202 | 0.079452 | 0 |
| TCGA-BP-4974 | 0.578082 | 1 |
| TCGA-CJ-4904 | 4.909589 | 0 |
| TCGA-B2-3923 | 0.991781 | 0 |
| TCGA-B0-5711 | 8.030137 | 0 |
| TCGA-BP-4770 | 0.90137 | 1 |
| TCGA-CJ-4881 | 5.517808 | 0 |
| TCGA-BP-4327 | 0.29863 | 1 |
| TCGA-B0-4842 | 4.723288 | 1 |
| TCGA-CZ-5470 | 0 | 0 |
| TCGA-CJ-4897 | 4.953425 | 0 |
| TCGA-BP-4165 | 8.320548 | 0 |
| TCGA-DV-5565 | 3.641096 | 0 |
| TCGA-B8-5550 | 1.189041 | 0 |
| TCGA-B8-4153 | 1.109589 | 0 |
| TCGA-BP-4174 | 5.147945 | 0 |
| TCGA-B0-4824 | 4.539726 | 1 |
| TCGA-CJ-4902 | 4.164384 | 0 |
| TCGA-EU-5907 | 0.347945 | 0 |
| TCGA-CZ-4857 | 3.923288 | 1 |
| TCGA-B0-5098 | 4.339726 | 1 |
| TCGA-BP-5004 | 3.084932 | 0 |
| TCGA-A3-3319 | 3.09589 | 0 |
| TCGA-BP-4167 | 7.446575 | 0 |
| TCGA-B0-5709 | 8.539726 | 0 |
| TCGA-BP-4982 | 2.778082 | 0 |
| TCGA-CJ-5672 | 5.40274 | 1 |
| TCGA-B0-5695 | 3.890411 | 0 |
| TCGA-B0-4818 | 1.39726 | 1 |
| TCGA-AK-3436 | 5.6 | 0 |
| TCGA-CZ-5469 | 2.591781 | 1 |
| TCGA-CZ-5984 | 4.084932 | 0 |
| TCGA-B2-3924 | 1.016438 | 0 |
| TCGA-B0-5088 | 1.542466 | 1 |
| TCGA-BP-5184 | 3.10411 | 0 |
| TCGA-BP-5200 | 2.912329 | 0 |
| TCGA-BP-5177 | 0.80274 | 0 |
| TCGA-B0-4698 | 0.115068 | 1 |
| TCGA-CJ-5671 | 5.323288 | 0 |
| TCGA-B8-5549 | 0.531507 | 0 |
| TCGA-BP-4776 | 1.126027 | 0 |
| TCGA-CZ-4856 | 0.049315 | 0 |
| TCGA-A3-3359 | 6.860274 | 0 |
| TCGA-BP-4994 | 3.583562 | 0 |
| TCGA-B2-4101 | 0.515068 | 0 |
| TCGA-BP-5185 | 3.10137 | 0 |
| TCGA-CJ-4868 | 1.769863 | 1 |
| TCGA-A3-3376 | 4.646575 | 1 |
| TCGA-B0-4710 | 0.263014 | 0 |
| TCGA-B0-5106 | 4.378082 | 1 |
| TCGA-B8-5159 | 0.657534 | 0 |
| TCGA-CJ-4870 | 4.10411 | 0 |
| TCGA-A3-3363 | 0.873973 | 0 |
| TCGA-B2-4098 | 0.139726 | 1 |
| TCGA-CZ-5987 | 1.219178 | 1 |
| TCGA-DV-5569 | 0.972603 | 0 |
| TCGA-BP-5181 | 4.09589 | 0 |
| TCGA-B0-5075 | 1.745205 | 1 |
| TCGA-B0-5399 | 1.786301 | 0 |
| TCGA-CJ-4644 | 0.920548 | 1 |
| TCGA-CZ-5466 | 1.876712 | 0 |
| TCGA-A3-3316 | 4.090411 | 0 |
| TCGA-BP-4762 | 3.679452 | 1 |
| TCGA-CJ-5675 | 6.657534 | 0 |
| TCGA-BP-4170 | 6.419178 | 1 |
| TCGA-B0-4690 | 0.117808 | 1 |
| TCGA-B0-5705 | 10.04932 | 0 |
| TCGA-A3-3372 | 2.013699 | 0 |
| TCGA-A3-3324 | 3.249315 | 0 |
| TCGA-BP-4970 | 1.186301 | 0 |
| TCGA-CJ-4901 | 3.972603 | 0 |
| TCGA-DV-5576 | 1.991781 | 1 |
| TCGA-BP-4983 | 3.871233 | 0 |
| TCGA-CJ-4869 | 6.99726 | 0 |
| TCGA-BP-4337 | 0.005479 | 1 |
| TCGA-CJ-4895 | 3.287671 | 1 |
| TCGA-B0-5104 | 0 | 1 |
| TCGA-BP-4758 | 6.049315 | 0 |
| TCGA-CZ-5458 | 4.268493 | 0 |
| TCGA-BP-4972 | 4.115068 | 0 |
| TCGA-B8-A54J | 1.446575 | 0 |
| TCGA-CJ-4891 | 2.243836 | 1 |
| TCGA-BP-4803 | 0.558904 | 0 |
| TCGA-DV-5566 | 3.830137 | 0 |
| TCGA-CJ-4878 | 5.989041 | 0 |
| TCGA-CZ-4854 | 3.846575 | 1 |
| TCGA-BP-4991 | 3.871233 | 0 |
| TCGA-CZ-5989 | 4.380822 | 0 |
| TCGA-CZ-4865 | 0.454795 | 1 |
| TCGA-B0-5402 | 1.230137 | 0 |
| TCGA-A3-3328 | 3.794521 | 0 |
| TCGA-3Z-A93Z | 1.054795 | 0 |
| TCGA-B0-4815 | 4.350685 | 1 |
| TCGA-A3-3320 | 4.131507 | 0 |
| TCGA-B2-5636 | 0.726027 | 0 |
| TCGA-BP-4986 | 2.150685 | 0 |
| TCGA-CZ-5460 | 3.917808 | 0 |
| TCGA-B4-5844 | 0.019178 | 0 |
| TCGA-B0-5120 | 1.350685 | 0 |
| TCGA-BP-4998 | 2.553425 | 0 |
| TCGA-AK-3455 | 1.871233 | 1 |
| TCGA-CJ-5682 | 5.158904 | 0 |
| TCGA-B4-5836 | 0.386301 | 0 |
| TCGA-B8-5165 | 0.021918 | 0 |
| TCGA-B0-5107 | 2.539726 | 1 |
| TCGA-B0-5099 | 1.328767 | 1 |
| TCGA-B0-4821 | 3.369863 | 1 |
| TCGA-B0-5119 | 0.161644 | 0 |
| TCGA-BP-4354 | 2.832877 | 1 |
| TCGA-B8-5552 | 1.073973 | 0 |
| TCGA-A3-3322 | 4.049315 | 0 |
| TCGA-BP-4959 | 7.287671 | 0 |
| TCGA-B8-4151 | 0.767123 | 0 |
| TCGA-BP-5194 | 1.117808 | 0 |
| TCGA-CJ-4884 | 4.819178 | 0 |
| TCGA-CZ-5461 | 0.90411 | 1 |
| TCGA-B4-5378 | 0.479452 | 0 |
| TCGA-B0-4697 | 1.583562 | 1 |
| TCGA-CJ-4874 | 6.254795 | 0 |
| TCGA-AK-3453 | 3.827397 | 0 |
| TCGA-BP-4353 | 1.027397 | 1 |
| TCGA-CJ-4637 | 6.10137 | 1 |
| TCGA-CJ-5683 | 5.175342 | 0 |
| TCGA-B0-5698 | 7.076712 | 0 |
| TCGA-CJ-5678 | 1.572603 | 1 |
| TCGA-BP-4342 | 6.180822 | 1 |
| TCGA-BP-4334 | 1.767123 | 1 |
| TCGA-BP-4761 | 0.49863 | 0 |
| TCGA-B4-5843 | 0.030137 | 0 |
| TCGA-BP-4355 | 2.610959 | 1 |
| TCGA-B0-4813 | 0.049315 | 1 |
| TCGA-AK-3451 | 4.057534 | 0 |
| TCGA-B2-5635 | 0.863014 | 0 |
| TCGA-CW-5589 | 6.515068 | 0 |
| TCGA-B8-5553 | 1.191781 | 0 |
| TCGA-B0-4841 | 0.558904 | 1 |
| TCGA-CZ-5985 | 4.463014 | 0 |
| TCGA-CW-5584 | 0.449315 | 1 |
| TCGA-CJ-5680 | 2.10411 | 1 |
| TCGA-B0-5116 | 1.8 | 0 |
| TCGA-BP-4329 | 2.315068 | 1 |
| TCGA-A3-A8OU | 0 | 0 |
| TCGA-B0-5707 | 7.747945 | 0 |
| TCGA-CZ-5457 | 4.238356 | 0 |
| TCGA-CJ-4885 | 5.821918 | 0 |
| TCGA-CJ-4639 | 6.323288 | 0 |
| TCGA-CW-5587 | 6.09863 | 0 |
| TCGA-B4-5832 | 0.424658 | 0 |
| TCGA-B0-5097 | 1.821918 | 0 |
| TCGA-G6-A8L7 | 5.843836 | 0 |
| TCGA-CZ-4866 | 4.843836 | 0 |
| TCGA-DV-A4VX | 4.454795 | 1 |
| TCGA-B0-5094 | 0.912329 | 1 |
| TCGA-BP-4164 | 2.717808 | 1 |
| TCGA-B8-A54G | 0.145205 | 0 |
| TCGA-CJ-4889 | 5.331507 | 0 |
| TCGA-BP-4775 | 5.049315 | 0 |
| TCGA-B8-A54E | 2.490411 | 0 |
| TCGA-CW-6097 | 1.564384 | 1 |
| TCGA-B0-4810 | 1.309589 | 1 |
| TCGA-A3-3313 | 2.013699 | 1 |
| TCGA-B0-5121 | 1.517808 | 0 |
| TCGA-CZ-5988 | 1.89863 | 0 |
| TCGA-BP-4782 | 0.969863 | 0 |
| TCGA-BP-4977 | 1.243836 | 0 |
| TCGA-BP-4759 | 6.49863 | 0 |
| TCGA-CJ-5677 | 2.142466 | 1 |
| TCGA-B0-5812 | 8.117808 | 0 |
| TCGA-CW-5585 | 7.147945 | 0 |
| TCGA-A3-3358 | 3.580822 | 0 |
| TCGA-B0-4836 | 3.391781 | 1 |
| TCGA-BP-5169 | 0.528767 | 0 |
| TCGA-G6-A8L6 | 0.857534 | 1 |
| TCGA-BP-4976 | 4.471233 | 0 |
| TCGA-CZ-5462 | 0.852055 | 1 |
| TCGA-CJ-5686 | 5.583562 | 0 |
| TCGA-BP-4781 | 5.69863 | 0 |
| TCGA-BP-4971 | 4.073973 | 0 |
| TCGA-BP-5192 | 1.956164 | 0 |
| TCGA-B0-4839 | 4.490411 | 1 |
| TCGA-CJ-4890 | 5.712329 | 0 |
| TCGA-BP-4964 | 5.10137 | 0 |
| TCGA-BP-5010 | 2.405479 | 1 |
| TCGA-BP-4768 | 1.09589 | 0 |
| TCGA-CJ-4905 | 4.09863 | 0 |
| TCGA-BP-4177 | 4.575342 | 0 |
| TCGA-BP-4326 | 4.452055 | 1 |
| TCGA-B2-5639 | 1.142466 | 0 |
| TCGA-A3-3306 | 3.068493 | 0 |
| TCGA-A3-A6NN | 0.008219 | 0 |
| TCGA-CZ-4862 | 5.049315 | 0 |
| TCGA-BP-4158 | 9.252055 | 0 |
| TCGA-B0-5690 | 6.59726 | 0 |
| TCGA-CJ-5689 | 0 | 1 |
| TCGA-MW-A4EC | 1.364384 | 0 |
| TCGA-AK-3461 | 2.336986 | 0 |
| TCGA-BP-4784 | 5.079452 | 0 |
| TCGA-B2-4102 | 0.553425 | 0 |
| TCGA-CZ-4858 | 5.323288 | 0 |
| TCGA-B0-4847 | 2.172603 | 1 |
| TCGA-BP-4344 | 4.564384 | 0 |
| TCGA-AK-3426 | 2.424658 | 1 |
| TCGA-BP-4341 | 4.353425 | 1 |
| TCGA-B8-A54K | 1.284932 | 0 |
| TCGA-CJ-4638 | 1.180822 | 1 |
| TCGA-CJ-4641 | 4.550685 | 1 |
| TCGA-B0-5085 | 2.109589 | 1 |
| TCGA-BP-4801 | 3.079452 | 0 |
| TCGA-B0-5693 | 8.427397 | 0 |
| TCGA-CJ-4918 | 0.254795 | 1 |
| TCGA-CZ-4853 | 2.120548 | 0 |
| TCGA-CW-6090 | 6.991781 | 0 |
| TCGA-BP-4338 | 7.832877 | 0 |
| TCGA-B0-5084 | 0.608219 | 1 |
| TCGA-B0-4712 | 3.663014 | 1 |
| TCGA-AK-3425 | 9.158904 | 0 |
| TCGA-BP-4789 | 4.079452 | 0 |
| TCGA-CZ-5465 | 3.772603 | 0 |
| TCGA-CJ-6028 | 4.452055 | 1 |
| TCGA-CW-6093 | 8.619178 | 0 |
| TCGA-B0-4713 | 0.553425 | 1 |
| TCGA-B0-4694 | 0.290411 | 1 |
| TCGA-BP-4993 | 0.484932 | 0 |
| TCGA-B0-4843 | 0.876712 | 1 |
| TCGA-BP-4799 | 3.10411 | 1 |
| TCGA-B0-4707 | 1.643836 | 1 |
| TCGA-A3-3382 | 1.572603 | 0 |
| TCGA-B0-5095 | 0.671233 | 1 |
| TCGA-B0-5115 | 2.183562 | 0 |
| TCGA-BP-5180 | 6.2 | 0 |
| TCGA-BP-5195 | 2.052055 | 0 |
| TCGA-CJ-4887 | 2.553425 | 1 |
| TCGA-BP-4965 | 5.126027 | 0 |
| TCGA-B0-4819 | 0.50137 | 1 |

Survival status: 0 represent alive, 1represent dead.
