# Supplementary material for: Identification of a m6A RNA methylation regulators-based signature for predicting the prognosis of clear cell renal carcinoma
Source: Cancer Cell Int. 2020 May 7;20:157. doi: 10.1186/s12935-020-01238-3 (PMC7206820; doi:10.1186/s12935-020-01238-3)
Supplement: Supplementary file 7 — Additional file 7: Table S3. Coefficients based on a multivariate Cox regression analysis. [file 12935_2020_1238_MOESM7_ESM.docx]

Table S3 Coefficients based on a multivariate Cox regression analysis.

| genes | coef | HR | 95% CI of HR | p value |
| --- | --- | --- | --- | --- |
| METTL14 | -0.385 | 0.681 | 0.546-0.848 | 0.0006 |
| METTL3 | 0.121 | 1.129 | 1.020-1.249 | 0.019 |

Abbreviation: HR, hazard ratio.
